# Synthesis, DFT Calculations, *In Silico* Studies, and Antimicrobial Evaluation of Benzimidazole-Thiadiazole Derivatives

**DOI:** 10.1021/acsomega.4c00543

**Published:** 2024-04-09

**Authors:** Ayşen Işık, Ulviye Acar Çevik, Arzu Karayel, Iqrar Ahmad, Harun Patel, İsmail Çelik, Ülküye Dudu Gül, Gizem Bayazıt, Hayrani Eren Bostancı, Ahmet Koçak, Yusuf Özkay, Zafer Asım Kaplancıklı

**Affiliations:** † Department of Biochemistry, Faculty of Science, Selçuk University, Konya, Turkey; ‡ Department of Pharmaceutical Chemistry, Faculty of Pharmacy, Anadolu University, Eskişehir 26470, Turkey; § Department of Physics, Faculty of Arts and Science, Hitit University, Çorum 19030, Turkey; ∥ Department of Pharmaceutical Chemistry, Prof. Ravindra Nikam College of Pharmacy, Gondur, Dhule, Maharashtra 424002, India; ⊥ Division of Computer Aided Drug Design, Department of Pharmaceutical Chemistry, R. C. Patel Institute of Pharmaceutical Education and Research, Shirpur, Maharashtra 425405, India; # Department of Pharmaceutical Chemistry, Faculty of Pharmacy, Erciyes University, Kayseri 38039, Turkey; ∇ Department of Bioengineering, Faculty of Engineering, Bilecik Seyh Edebali University, Bilecik, Turkey; ○ Department of Biotechnology, Institute of Graduate Studies, Bilecik Seyh Edebali University, Bilecik, Turkey; ◆ Department of Biochemistry, Faculty of Pharmacy, Cumhuriyet University, Sivas, Turkey; ¶ Department of Chemistry, Faculty of Science, Selçuk University, Konya, Turkey

## Abstract

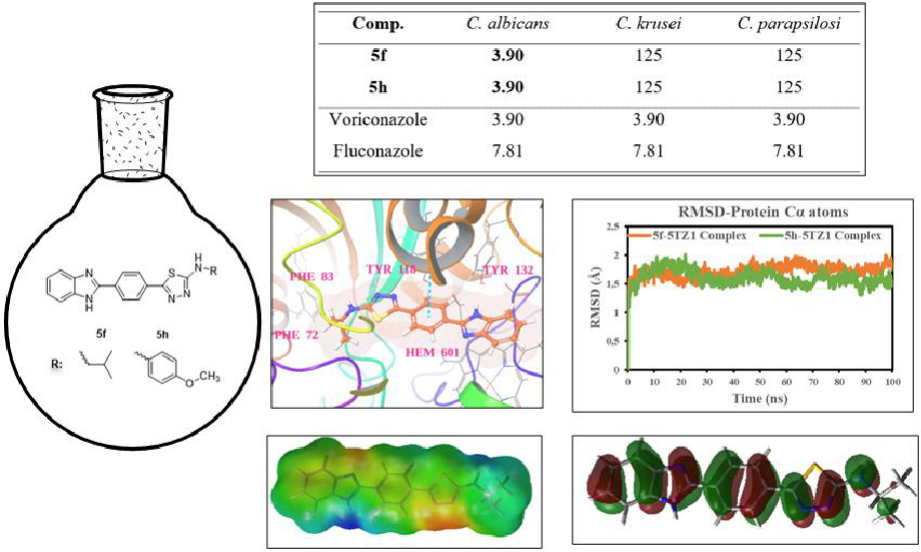

In this study, a series of new benzimidazole-thiadiazole hybrids were synthesized, and the synthesized compounds were screened for their antimicrobial activities against eight species of pathogenic bacteria and three fungal species. Azithromycin, voriconazole, and fluconazole were used as reference drugs in the mtt assay. Among them, compounds **5f** and **5h** showed potent antifungal activity against *C. albicans* with a MIC of 3.90 μg/mL. Further, the results of the antimicrobial assay for compounds **5a**, **5b**, **5f**, and **5h** proved to be potent against *E. faecalis* (ATCC 2942) on the basis of an acceptable MIC value of 3.90 μg/mL. The cytotoxic effects of compounds that are effective as a result of their antimicrobial activity on healthy mouse fibroblast cells (L929) were evaluated. According to HOMO–LUMO analysis, compound **5h** (with the lower Δ*E* = 3.417 eV) is chemically more reactive than the other molecules, which is compatible with the highest antibacterial and antifungal activity results. A molecular docking study was performed to understand their binding modes within the sterol 14-α demethylase active site and to interpret their promising fungal inhibitory activities. Molecular dynamics (MD) simulations of the most potent compounds **5f** and **5h** were found to be quite stable in the active site of the 14-α demethylase (5TZ1) protein.

## 1 Introduction

Antimicrobial resistance (AMR) is a significant public health concern of the twenty-first century. It threatens the effective prevention and treatment of an increasing variety of microbial diseases that are resistant to the traditional drugs used to treat them.^1^ Microbial resistance is one of the most burning problems in clinical practice, and one of the main objectives of current biomedical research is to discover new, powerful drugs that can combat multiresistant bacteria. Antibiotic abuse and pharmaceutical corporations’ lack of interest in investing in antibiotic development has made the discovery of new antibiotic classess inevitable.^2−4^

Benzimidazole is known as a lucky structure in pharmaceutical chemistry and has a variety of biological functions. Benzimidazole has a benzene ring system in which the benzene ring is attached to a five-member imidazole ring having nitrogen atoms at positions 1 and 3; thus it is known as a heterocyclic aromatic compound.^5,6^ Additionally, benzimidazole is a tremendous scaffold of therapeutic importance with promising pharmacological properties. The safety and efficacy profiles of benzimidazole medications in clinical use are well established. Because of their therapeutic effects, benzimidazole derivatives have attracted a lot of interest in the medical field, providing excellent results as anticancer,^7^ antiviral,^8^ antimicrobial, anti-inflammatory, antihistamine,^9^ antihypertensive, antitubercular, analgesics, antiulcer, and anthelmintics.^10^ The benzimidazole ring is present in many significant medications that are therapeutically employed in the research field. Albendazole (anthelminthic), Bendamustine (anticancer), Omeprazole (antiulser), and Astemizole (antihistamine) are examples of drugs with a benzimidazole structure.^11,12^

Thiadiazoles are five-membered heterocyclic compounds with two nitrogen atoms and one sulfur atom. They are a type of azole molecule.^13^ During the last decades, 1,3,4-thiadiazole derivatives have drawn much attention due to their biological and pharmaceutical activities and have been investigated increasingly due to their numerous therapeutic and industrial applications, which is due to the presence of =N–C–S- moiety.^14^ A variety of 1,3,4-thiadiazole is in use, like Acetazolamide (diuretic), Cefazolin, Cefazedone (antibiotics), Megazol (antiprotozoal), Timolol maleate (NSAIDs), Methazolamide (carbonic anhydrase inhibitor), and Sulphamethizole (antibacterial).^15^

In this study, a new series of benzimidazole-thiadiazole derivatives were synthesized and characterized by^1^H NMR, ^13^C NMR, and HRMS. Synthesized compounds were screened for their antimicrobial activities against eight species of pathogenic bacteria [*Escherichia coli* (ATCC 25 922), *Serratia marcescens* (ATCC 8100), *Klebsiella pneumoniae* (ATCC 13 883), *Pseudomonas aeruginosa* (ATCC 27 853), *Enterococcus faecalis* (ATCC 2942), *Bacillus subtilis*, *Staphylococcus. aureus* (ATCC 29 213), *S. epidermidis* (ATCC 12 228)] and three fungal species *Candida albicans* (ATCC 24 433), *C. krusei* (ATCC 6258), *C. parapsilosis* (ATCC 22 019)]. The cytotoxic effects of the final compounds that are effective as a result of antimicrobial activity on healthy mouse fibroblast cells (L929) were evaluated. With the use of *Candida*’s 14α-demethylase (CYP51), molecular docking investigations were also carried out. Using 100 ns molecular dynamics (MD) simulations, we investigated the stability of compounds containing CYP51 was investigated. It is crucial to know the precise structure of the ligand with the lowest minimum energy when conducting a molecular docking investigation. Density functional theory (DFT) was used to model the eight newly synthesized chemicals.

## 2 Results and Discussion

### 2.1 Chemistry

**Figure 1:**
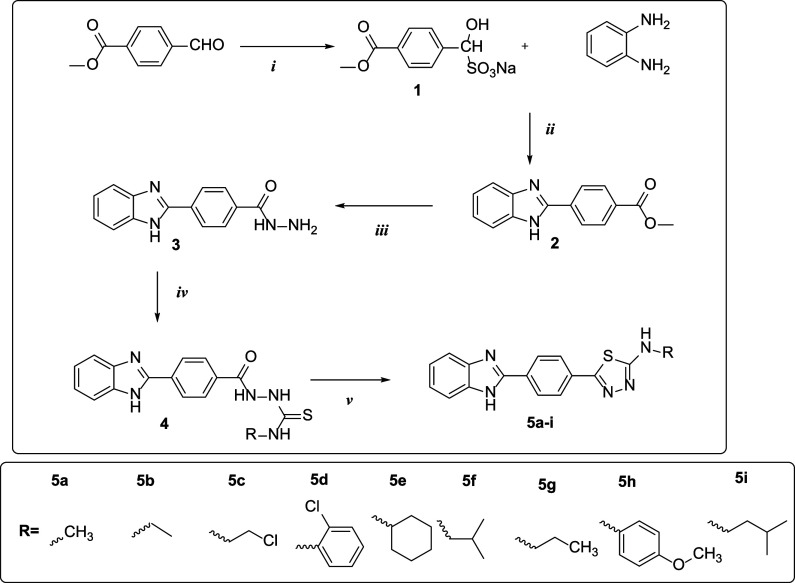
Synthesis pathway of 1,3,4-thiazole derivative target benzimidazole compounds 5a-5i. Reagent and conditions:(i)Na_2_S_2_O_5_/EtOH,(ii)DMF/120 °C,(iii)NH_2_NH_2_/EtOH,(iv)RNCS/EtOH, and (v) H_2_SO_4_

The target molecules were synthesized in five processes, as shown in Figure 1. To obtain the sodium metabisulfite addition product of the aldehyde, the methyl 4-formylbenzoate compound aldehyde was first treated with sodium metabisulfite in ethanol. In the second step, methyl 4-(1*H*-benzo[*d*]imidazole-2-yl)benzoate (**2**) was produced as a consequence of the condensation reaction between the sodium metabisulfite product and benzen-1,2-diamine under reflux. Compound **2** was treated with hydrazine hydrate in ethanol in the following step to produce compound (**3**). Ethanol was refluxed with the hydrazide derivative compound and the corresponding isothiocyanate derivatives, and the precipitated product was filtered out. The thiosemicarbazide molecule was cyclized in the presence of strong sulfuric acid to produce the thiadiazole derivatives (**5a**–**i**) in the last step.

### 2.2 Antimicrobial Activity

The antifungal and antibacterial activities of the synthesized compounds (**5a**–**i**) were evaluated *in vitro* against *E. coli* (ATCC 25922), *S. marcescens* (ATCC 8100), *K. pneumoniae* (ATCC 13883), *P. aeruginosa* (ATCC 27853), *E. faecalis* (ATCC 2942), *B. subtilis*, *S. aureus* (ATCC 29213), *S. epidermidis* (ATCC 12228), *C. albicans* (ATCC 24433), **C. krusei** (ATCC 6258), and *C. parapsilosis* (ATCC 22019). The final synthesized compounds were evaluated as antibacterial and antifungal references, in comparison to azithromycin, voriconazole, and fluconazole. Tables 1 and 2 provide a summary of the antimicrobial activity test results and the minimum inhibitory concentrations (MICs) of the compounds (**5a**–**i**).

**Table 1:** Antifungal Activity of the Compounds **5a**–**5i** as MIC Values (μg/mL)

comp.	*C. albicans*(ATCC 24433)	*C. krusei*(ATCC 6258)	*C. parapsilosis*(ATCC 22019)
**5a**	31.25	125	125
**5b**	**7.81**	31.25	15.625
**5c**	31.25	125	125
**5d**	**7.81**	125	125
**5e**	**7.81**	125	125
**5f**	**3.90**	125	125
**5g**	**7.81**	125	125
**5h**	**3.90**	125	125
**5i**	31.25	125	125
**voriconazole**	3.90	3.90	3.90
**fluconazole**	7.81	7.81	7.81

**Table 2:** Antibacterial Activity of the Compounds **5a**–**5i** as MIC Values (μg/mL)^a^

comp.	A	B	C	D	E	F	G	H
**5a**	250	62.5	125	62.5	**3.90**	125	**7.81**	**7.81**
**5b**	62.5	62.5	62.5	62.5	**3.90**	125	31.25	31.25
**5c**	62.5	62.5	62.5	125	15.625	62.5	31.25	31.25
**5d**	125	62.5	125	125	**7.81**	125	**7.81**	31.25
**5e**	125	125	125	125	15.625	250	125	**7.81**
**5f**	125	125	125	125	**3.90**	125	62.5	3.90
**5g**	125	250	125	125	15.625	125	31.25	31.25
**5h**	31.25	62.5	62.5	31.25	**3.90**	125	31.25	31.25
**5i**	125	62.5	62.5	31.25	15.625	125	62.5	62.5
**azithromycin**	<0.97	<0.97	<0.97	<0.97	<0.97	<0.97	<0.97	<0.97

a Most active compounds. A: *E. coli* (ATCC 25922), B: *S. marcescens* (ATCC 8100), C: *K. pneumoniae* (ATCC 13883), D: *P. aeruginosa* (ATCC 27853),E: *E. faecalis* (ATCC 2942), F: *B. subtili*s (ATCC ), G: *S. aureus* (ATCC 29213), H: *S. epidermidis* (ATCC 12228) A–D: Gram-negative bacteria, E–H: Gram-positive bacteria. S.D: Standard Drug = Azithromycin.

Among the series, compounds **5a**, **5b**, **5f**, and **5h** were found to be the most active molecules with an MIC value of 3.90 μg/mL, and they are specific toward the Gram-positive bacterial, *E. faecalis* (ATCC 2942). Compounds **5a** exhibited noteworthy activity with an MIC value of 7.81 μg/mL against *S. aureus* (ATCC 29213) and *S. epidermidis*. Compound **5d** exhibited noteworthy activity with an MIC value of 7.81 μg/mL against *S. aureus* (ATCC 29213) and *E. faecalis* (ATCC 2942). Compound **5e** (MIC 7.81 μg/mL) against *S. epidermidis* (ATCC 12228) exhibited comparable potency to the reference drug used.

When the antifungal activity test results of the compounds were examined, it was found that the compounds were generally more effective against *C. albicans*. According to the antifungal activity test, compounds **5f** and **5h** exhibited better potency with an MIC value 3.90 μg/mL against *C. albicans* comparable to the reference drug fluconazole (MIC 7.81 μg/mL) and voriconazole (MIC 3.90 μg/mL). Compounds **5b**, **5d**, **5e,** and **5g** were observed to have significant antifungal activity on *C. albicans*. The MIC values for these compounds were determined to be 7.81 μg/mL.

Structure–activity relationship (SAR), after closely reviewing the data for antibacterial activity, the following conclusions were reached:

- Activity data revealed that compared to Gram-negative bacteria, the majority of the synthesized benzimidazole-thiadiazole derivatives shown superior potency against Gram-positive bacteria.
- In most of the cases, ethyl substituted showed better antibacterial inhibitory activity than 2-chloroethyl substituted against **E. faecalis** (ATCC 2942).
- *N*-isopropyl substituted showed better antibacterial inhibitory activity than *N*-propyl and *N*-isobutyl substituted.
- It was determined that the synthesized compounds were more sensitive to **C. albicans**.

### 2.3 Cytotoxicity Assay

**Figure 2:**
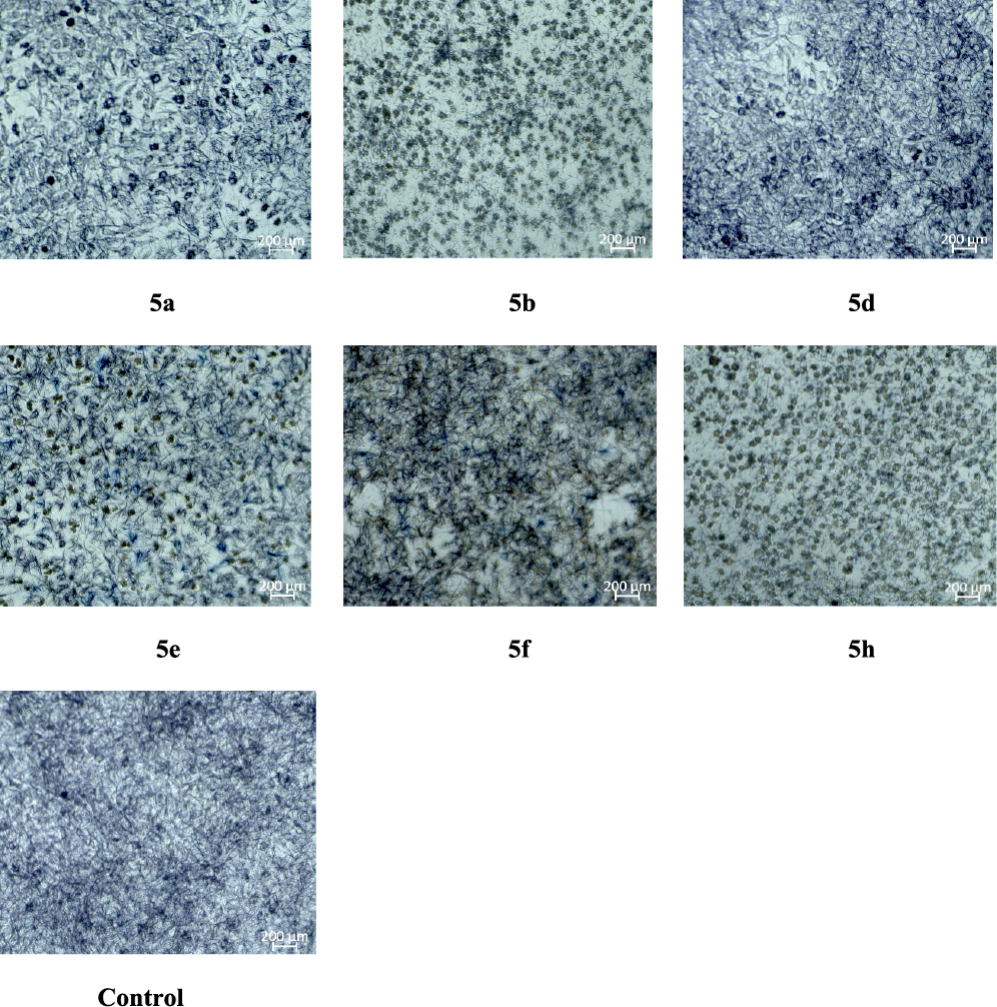
Cell line views for all compounds **5a**, **5b**, **5d**, **5e**, **5f**, and **5h** and control.

In order to evaluate the cytotoxic effects of compounds **(5a, 5b, 5d, 5e, 5f, 5h)** on healthy cells, the compounds that are effective as a result of antimicrobial activity were selected and their cytotoxic effects on healthy mouse fibroblast cells (L929) were evaluated. The cell line appearances for all compounds are shown in Figure 2. The IC_50_ values of the compounds are given in Table 3.

**Table 3:** IC_50_ Values (μM) for L929 Fibroblast Cell Line

compounds	**IC**_**50**_**(μM)**
**5a**	152.02 ± 3.64
**5b**	118.78 ± 9.64
**5d**	196.82 ± 14.66
**5e**	101.82 ± 12.01
**5f**	174.84 ± 3.02
**5h**	75.96 ± 9.4

As a result of cytotoxicity studies conducted on healthy cell cultures, the IC_50_ values of all compounds except compound ″5h″ were found to be higher than 100 μM. Considering the MIC values of the compounds in antimicrobial activity, it is seen that they are much lower than the IC_50_ values at which the compounds have a toxic effect on healthy cells. From this information, it is concluded that the compounds show antimicrobial activity and are not toxic at the MIC values.

### 2.4 *In Silico* Studies

#### 2.4.1 Quantum Chemical Calculations

**Figure 3:**
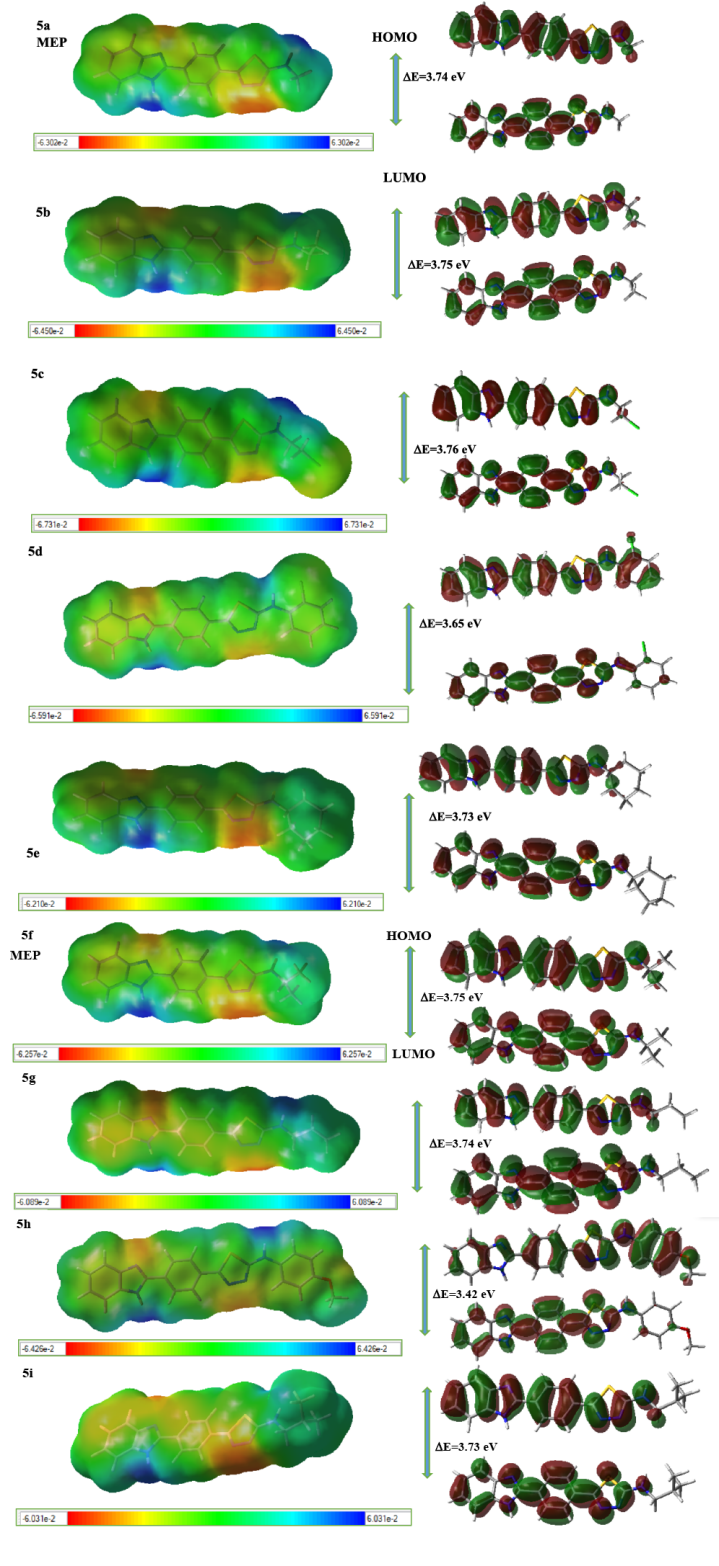
Molecular Electrostatic Potential (MEP) and HOMO–LUMO diagrams of compounds **5a**–**i** at the B3LYP/6-311G(d,p) level. Atom colors: carbon in gray, nitrogen in blue, chlorine in green, oxygen in red, sulfur in yellow, and hydrogen in white. The surfaces plotted by the 0.0004 electrons/b3 contour of the electronic density. For **5a** molecule: Color ranges: in Au: blue, more positive than 0.0630; green, between 0.0630 and 0; yellow, between 0 and – 0.0630; red, more negative than – 0.0630.

The density functional theory (DFT) calculations were performed by using the Gaussian 09 program^16^ with the B3LYP exchange correlation functional with the 6-311G(d,p) basis set. GaussView 5.0 program^17^ was used to generate the input geometries and visualize the results. The optimized geometries of all structures correspond to true minima as no imaginary frequencies are observed in the vibration frequency investigation. The molecular electrostatic potential (MEP) and HOMO–LUMO analyses were performed at the B3LYP/6-311G(d,p) level in order to investigate the electronic properties of current molecules. MEP and HOMO–LUMO diagrams are shown in Figure 3.

The molecular geometry parameters regarding the optimization results of all molecules are in good agreement with the experimental studies possessing similar main building blocks in the literature.^18^ The main molecule in the building blocks of compound **5h** consists of a 1, 3, 4-thiadiazole ring with an amine group attached to a methoxy-phenyl ring and a phenyl-benzimidazole group at the second and fifth locations of this ring, respectively. N–C single and *N* =C double bonds of benzimidazole ring are 1.379 and 1.316 Å, respectively, which are in line with the experimental values of 1.381 (3) Å and 1.324 (3) Å in literature.^18^ The rotation of the phenyl-benzimidazole group with respect to the 1, 3, 4-thiadiazole ring is measured as −179.6°, while the rotation of amine group attached to methoxy-phenyl ring to this ring is 180.0°. These values indicate that the molecule is planar. All molecules showed the same trend.

According to the HOMO–LUMO analysis, **5h** is chemically more reactive than the other molecules (Table 4**)**. The chemical reactivity order is **5***h***> 5d > 5***i***> 5e > 5***g***> 5a > 5***f***> 5b > 5c. 5c,** having the higher energy gap value (Δ*E* = 3.756 eV), is more stable and less reactive than the others, which is in line with the lowest antibacterial and antifungal activity results.

**Table 4:** HOMO–LUMO Energies (eV) and Calculated Global Reactivity Parameters of the Best Stable States of Compounds **5a**–**i** at the B3LYP/6-311G(d,p) Level in the Gas Phase^a^

compound	LUMO	HOMO	**Δ***E***(eV)**	IP (eV)	EA (eV)	**χ (eV)**	η (eV)	**σ (eV)**^**–1**^	μ (eV)	ω (eV)
**5a**	–1.939	–5.680	3.741	5.680	1.939	3.809	1.871	0.267	–3.809	3.879
**5b**	–1.961	–5.711	3.750	5.711	1.961	3.836	1.875	0.267	–3.836	3.924
**5c**	–2.111	–5.867	**3.756**	**5.867**	2.111	3.989	1.878	0.266	–3.989	4.236
**5d**	–2.192	–5.844	3.652	5.844	2.192	4.018	1.826	0.274	–4.018	4.421
**5e**	–1.941	–5.675	3.734	5.675	1.941	3.808	1.867	0.268	–3.808	3.883
**5f**	–1.952	–5.701	3.749	5.701	1.952	3.827	1.874	0.267	–3.827	3.906
**5g**	–1.915	–5.651	3.736	5.651	1.915	3.783	1.868	0.268	–3.783	3.831
**5h**	–2.063	–5.480	**3.417**	**5.480**	2.063	3.772	1.709	0.293	–3.772	4.162
**5i**	–1.912	–5.644	3.732	5.644	1.912	3.778	1.866	0.268	–3.778	3.824

a Δ*E* (*E*_LUMO_-*E*_HOMO_): band gap, IP (−HOMO): ionization potential, EA (−LUMO): electron affinity χ (IP+EA)/2: electronegativity, η (IP–EA)/2: chemical hardness, σ (1/2η): chemical softness,μ (−(IP+EA)/2): chemical potential, ω (μ^2^/2η): electrophilic index.

As known from the literature, a lower value of ionization potential (IP) indicates that it has a better property of electron donor.^19^ According to Table 4, it is seen that the ionization potential of **5h** is the lowest than the others, which refers to a better property of electron donor. A good description of donor–acceptor properties sheds light on hydrogen bonding interactions. This context provides a preliminary assessment for ligand-protein interactions. The electrophilic indexes (ω) of all molecules belong to the strong electrophiles group as their values are bigger than 1.50 eV.^20^

LUMOs are distributed throughout the whole molecule throughout the conjugated π system, while HOMOs are localized in electronegative nitrogen atoms and aromatic rings, as shown in Figure 3. In the **5h** molecule, since the methoxy group resonated with the phenyl ring, the electron density of the ring increased, and HOMO is localized in this part, while LUMO distribution is located in aromatic rings except the methoxy phenyl ring.

According to the MEP diagram (Figure 3), in all molecules, negative regions possessing the high electron density are seen around the N atoms in the benzimidazole and thiadiazole ring, which are responsible for electrophilic attacks. In addition to these negative regions, **5h** has also a negative zone around the oxygen of methoxy. Positive regions with the low electron density of all molecules are formed around both the N–H group in the benzimidazole ring and N–H group bonded to the thiadiazole ring, which are responsible for nucleophilic attacks.

#### 2.4.2 Molecular Docking Studies

Molecular docking studies of synthesized thiadiazole derivatives (**5a**–**i**) were performed using Glide (Grid-based Ligand Docking with Energetics) to understand their binding modes within the sterol 14-α demethylase active site and to interpret their promising fungal inhibitory activities. This target was chosen since it has been noted that mostly antifungals inhibit the enzyme lanosterol 14- α – demethylase, which is dependent on cytochrome P450 and depletes Ergosterol, a crucial component of the fungal cell membrane. Table 5(21,22)

**Table 5:** Glide Docking Score (kcal/mol) of Synthesized Compounds in the 14-α Demethylase (CYP51) from *C. albicans* (PDB ID: 5TZ1)

compounds	docking score
**5a**	–8.401
**5b**	–8.368
**5c**	–8.441
**5d**	–9.15
**5f**	–8.25
**5g**	–8.209
**5h**	–9.077
**5i**	–8.528
**VT1161**	–8.528
**fluconazole**	–6.633
**voriconazole**	–7.004

**Figure 4:**
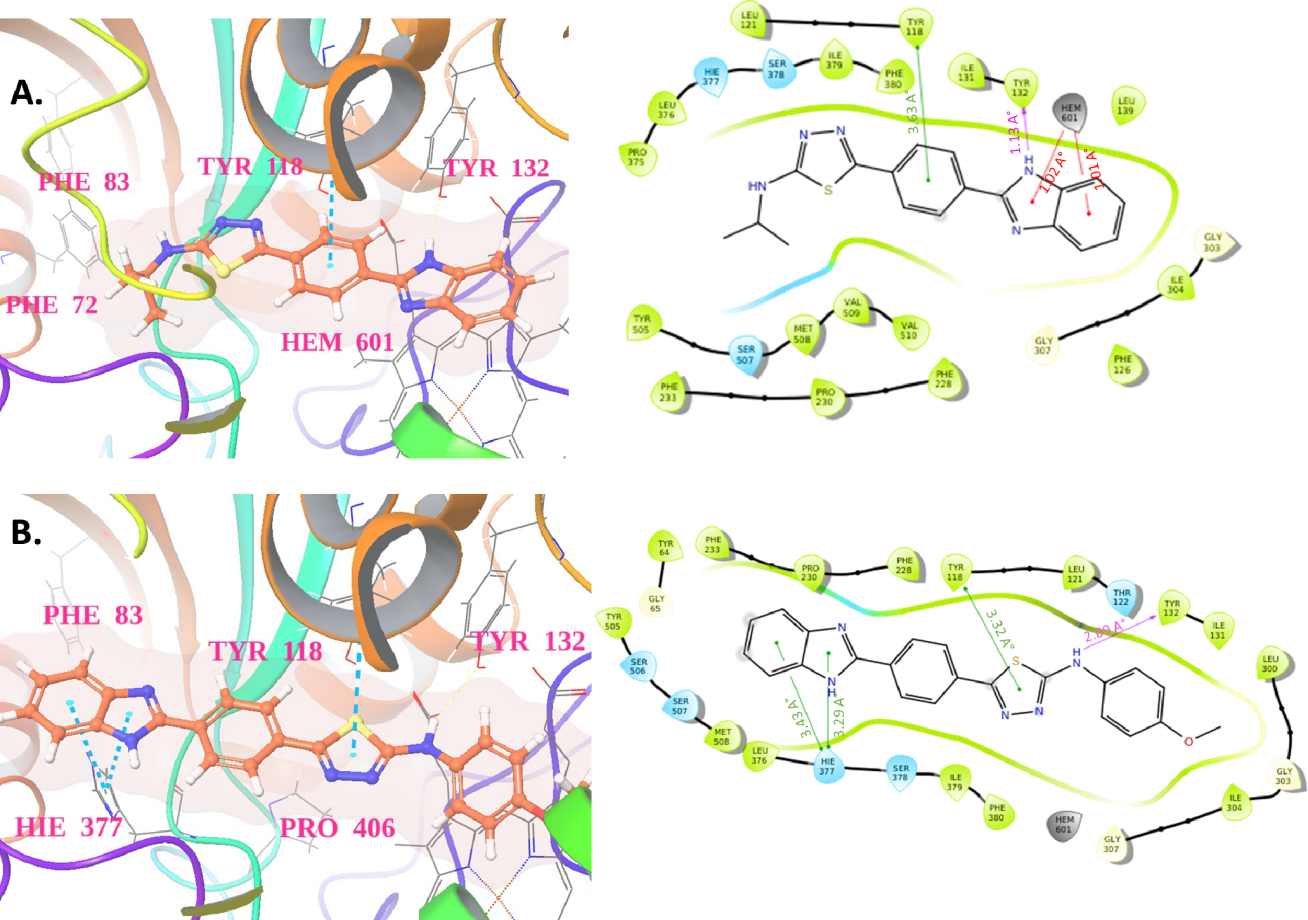
2D and 3D Binding interaction of Representative compound 5f and 5h in the active site of 14-α demethylase (CYP51) from *C. albicans* (PDB ID: 5TZ1).

It was observed that all the synthesized compounds nicely docked into the active site of *C. albicans* sterol 14-α demethylase with a good docking score ranging from −9.15 to −8.209 kcal/mol.^23,24^ According to the docking result analysis, the most active molecules **5f** (MIC= 3.90 μg/mL) and **5h** (MIC= 3.90 μg/mL) were effective against *C. albicans* and had significant binding affinity toward the 14-α demethylase protein with docking scores of −8.25 and −9.077 kcal/mol, respectively. The standard drugs Voriconazole, Fluconazole, and the cocrystallized inhibitor VT1161 had docking scores of −7.004, −6.633, and −8.528 kcal/mol, respectively. In previous studies and X-ray crystallographic structures, the binding pocket of VT1161 was identified with residues, such as Tyr64, Tyr118, Leu121, Thr122, Phe126, Ile131, Tyr132, Phe228, Pro230, Phe233, Gly303, Ile304, Gly307, Gly308, Thr311, Leu376, His377, Ser378, Phe380, Tyr505, Ser507, and Met508. Our docking results of synthesized thiadiazole derivatives with the 14-α demethylase protein also showed a similar docking profile. The 2D and 3D visual representations of the representative compound **5f** and **5h** interactions depicted in Figure 4 were generated using Maestro’s ligand interaction tool. Both of these compounds lodge in the active site with a similar approach to cocrystallized ligands and show hydrophobic and hydrogen bonding with different crucial residues. The binding interaction of compound **5f** shows that it forms hydrogen bonds with Tyr132 and ionic interactions with Hem 601 through the benzimidazole scaffold. At a distance of 3.63 Å, Tyr118 tacking with the central hydrophobic phenyl ring of compound **5f** (Figure 4A). In the case of compound **5h,** three hydrophobic interactions are visible with the benzimidazole (His377) and thiadiazole (Tyr1188) scaffolds and one hydrogen bond with Tyr132 in the active site of the 14-α demethylase protein (Figure 4B). The tetrazole-based antifungal drug candidate VT1161 demonstrates significant interactions with key residues within the 14-α demethylase protein, notably, Tyr118, Tyr132, and His377. Specifically, representative compounds **5f** and **5h** establish contact with these critical residues, underscoring their importance in the antifungal mechanism. The comparable docking scores of these compounds further suggest robust binding affinity, reinforcing the hypothesis that they exert antifungal effects by effectively suppressing the activity of the 14-α demethylase protein. This interaction profile highlights the potential of compounds **5f** and **5h** as promising candidates for further exploration in the development of antifungal therapeutics targeting *C. albicans*.

#### 2.4.3 Result Obtained from Molecular Dynamics Simulation

**Figure 5:**
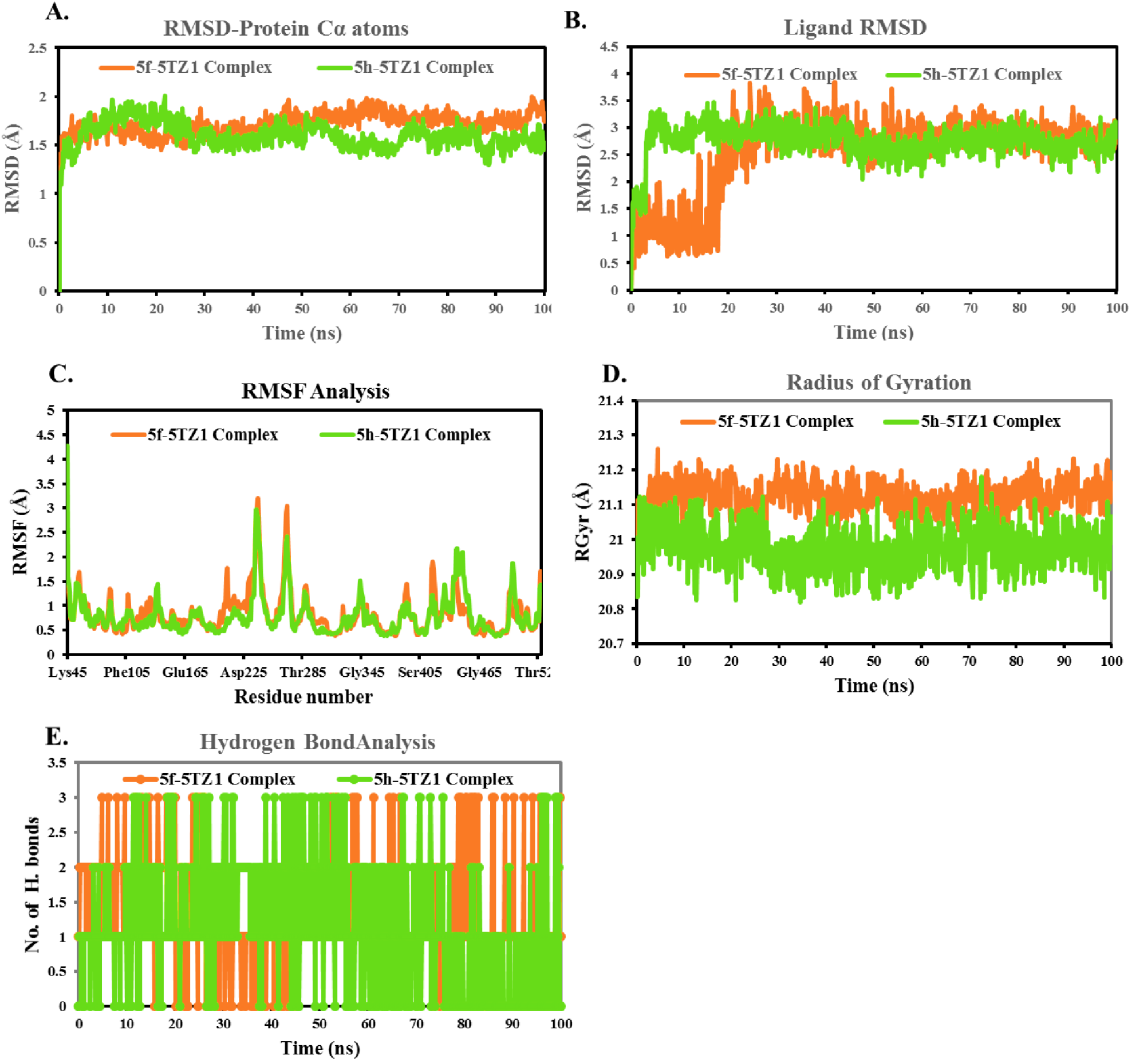
MD simulation trajectory analysis of ligand–protein complexes. A. Time-dependent RMSD plot; B. ligand RMSD is C. individual amino acids RMSF plot; D. time-dependent radius of gyration plot; E. time-dependent hydrogen bond analysis.

MD simulation for compounds **5f** and **5h** in complex with 14-α demethylase (5TZ1) protein selected for MD simulations along a time scale of 100 ns to validate the results of molecular docking and assess their conformational stability. The RMSD of α carbon atoms in all systems is analyzed to understand their stability. The RMSD of the **5f-**5TZ1 complex reaches to ∼1.73 Å from 0 to 50 ns, and after that, the system maintains the average RMSD value of 1.77 Å until the end of the simulation (Figure 5A). While assessing the RMSD of the **5h-**5TZ1 complex, a steady decrease in MSD is observed after 15 ns, and after 51 ns until 100 ns, a slight fluctuation is observed, indicating that the trajectories generated during these times are stable. The average RMSD for **5f** is 1.71 Å, whereas the RMSD for **5h** is detected around 1.60 Å, indicating higher structural stability in both complexes. The average ligand RMSD values for 5f and the 5h are 2.54 and 2.76 Å, respectively. For the 5f-5TZ1 complex, the ligand RMSD shows an initial lower value until 20 ns, after which small fluctuations occur. The RMSD remains consistent overall, indicating minor adaptations or adjustments in the ligand conformation during the simulation. On the other hand, in the case of the 5h-5TZ1 complex, the ligand RMSD exhibits no major fluctuations throughout the simulation, except for the initial time fluctuations attributed to equilibration. This suggests that the conformation of the 5h ligand remains stable and undergoes fewer structural changes compared to those of the 5f ligand during the entire simulation period (Figure 5B).

RMSF values are also calculated to determine the dynamic behavior of both protein residues. Maximum fluctuations of 3.20 and 3.04 Å are detected in residues Leu239 and Asp269, respectively. The visual analysis of MD simulation trajectories suggests that all the drugs engaged in significant binding interactions with the hotspot residues, namely, Gly307, Ile131, Leu121, Leu376, Leu87, Met508, Phe126, Phe233, Phe380, Pro230, Ser378, Tyr118, and Tyr132 of the 14-α demethylase protein. It was found that all of these interacting residues had RMSF values of 0.411 and 1.628 Å, indicating the stability of compounds **5f** and **5h** during simulation (Figure 5C). Less fluctuation is indicative of a stronger protein structural stability. The structural compactness of both complexes is analyzed by using the radius of gyration (RGyr). The radius of gyration is the estimated distance that a body’s mass is concentrated around an axis. It is frequently used to learn more about the conformational and folding behaviors of macromolecules.^23,24^ It is an evaluation of a macromolecule’s overall compactness and 3-D structure under diverse settings. The wide range of RGyr values indicated that the simulation may have caused the protein to unfold. RGyr is found in the range of 21.004–21.232 and 20.820–21.116 Å for 5f–5TZ1 and 5h–5TZ1 complexes, respectively, which indicates that the 14-α demethylase protein is almost stable during the whole run (Figure 5D).

In a protein–ligand complex, the strength of a ligand molecule’s binding to the target protein is determined by the presence of hydrogen bonds.

Compounds **5f** (orange) and **5h** (green) show significant intermolecular hydrogen bonding with the 14-α demethylase protein in the MD simulation at 100 ns, with a range from 0 to 3. The quantity and distribution of hydrogen bonds are displayed in Figure 5D. The maximum and mean hydrogen bond numbers of the 3a: CDK5A1 complex, **5f**–5TZ1 complex, and **5h**–5TZ1 complex were 3, 3, and 1.1 and 0.7, respectively (Figure 5E). Hydrogen bond analysis not only confirms the docking results but also shows that no conformational changes occurred in binding positions during the MD simulation.

## 3 Conclusion

In summary, a class of novel benzimidazole-1,3,4-thiadiazole hybrids was designed and synthesized and evaluated for antimicrobial activity. Activity data disclosed that most of the synthesized benzimidazole-thiadiazole derivatives showed better potency against Gram-positive bacteria as compared to Gram-negative bacteria. When the antifungal activity test results of the compounds were examined, it was found that the compounds were generally more effective against *C. albicans*. Compounds **5a**, **5b**, **5f**, and **5h** were found to be the most active molecules with MIC value 3.90 μg/mL against *E. faecalis* (ATCC 2942). According to the antifungal activity test, compounds **5f** and **5h** exhibited better potency with an MIC value of 3.90 μg/mL against *C. albicans*. According to the HOMO–LUMO analysis, compound **5h** (with the lower Δ*E* = 3.417 eV) is chemically more reactive than the other molecules, which is compatible with the highest antibacterial and antifungal activity results. The effects of the compounds on the L929 mouse fibroblast (normal) cell line were studied to determine the site of cytotoxicity. Furthermore, molecular docking studies are used to predict how designed or synthesized compounds interact with the target protein/enzyme. Molecular dynamics (MD) simulation of the most potent compounds **5f** and **5h** were found to be quite stable in the active site of the 14-α demethylase (5TZ1) protein.

## 4 Material and Method

The complete chemicals used in the synthetic process were acquired from Merck Chemicals (Merck KGaA, Darmstadt, Germany) or Sigma-Aldrich Chemicals (Sigma-Aldrich Corp., St. Louis, MO, USA). The compounds’ uncorrected melting points were ascertained using an MP90 digital melting point instrument (Mettler Toledo, OH, USA). A Bruker digital FT-NMR spectrometer (Bruker Bioscience, Billerica, MA, USA) operating at 300 and 75 MHz registered the^1^H- and^13^C NMR spectra of the compounds produced in DMSO-*d*_6_, respectively. In the NMR spectra, splitting patterns were denoted as follows: s for singlet, d for doublet, t for triplet, and m for multiplet. The reported unit of coupling constants (J) was Hertz. The Shimadzu LC/MSMS system (Shimadzu, Tokyo, Japan) was used to determine the M + 1 peaks. Using Silica Gel 60 F254 TLC for thin-layer chromatography (TLC), all reactions were observed.

### 4.1 Chemistry

#### 4.1.1 Synthesis of Sodium Metabisulfite Salt of Benzaldehyde (1) Derivative

Ethanol was used to dissolve methyl 4-formyl benzoate (5g, 0.03 mol). Drop by drop, sodium metabisulfite (6.84 g, 0.036 mol) in ethanol was added to the benzaldehyde solution. The reaction mixture was agitated for 1 h at room temperature following the completion of the dripping process. The precipitated product was removed by using a filter.

#### 4.1.2 Synthesis of Methyl 4-(1H-Benzo[d]imidazole-2-Yl)benzoate (2)

After dissolving benzen-1,2-diamine (0.022 mol) in DMF, sodium metabisulfite salt of benzaldehyde derivative (7.09 g, 0.026 mol) was added. Pouring the reaction contents into freezing water at the end of the reaction allowed the result to precipitate. After being filtered out, the precipitated product crystallized from the ethanol.

#### 4.1.3 Synthesis of 4-(1H-Benzimidazole-2-Yl)benzohydrazide Derivatives (3)

Compound 2 (0.018 mol) was added to the same vial, along with an excess of hydrazine hydrate (5 mL) and 15 mL of ethanol. For 12 h, the mixture was refluxed. Following the completion of the reaction, the mixture was placed in freezing water, and the result was filtered.

#### 4.1.4 *N*-Substituted-2-(4-(1H-Benzimidazole-2-Yl)benzoyl)hydrazine-1-Carbothioamide (4)

Compound **3** (1 mmol) and the appropriate isothiocyanate (1.1 mmol) were dissolved in 10 mL of ethanol and refluxed for 3 h. The precipitated product was filtered off.

#### 4.1.5 *N*-Substituted-5-(4-(1H-Benzimidazole-2-Yl)phenyl)-1,3,4-Thiadiazole-2-Amine (5a–5i)

The appropriate thiosemicarbazide derivative was stirred in 10 mL of H_2_SO_4_ in an ice bath. Then, it was stirred for another 10 min at room temperature, at the end of the time, it was poured slowly on ice, adjusted to pH = 8 with aqueous ammonia, and filtered. It is washed with water and crystallized from ethanol.

#### 4.1.6 *N*-Methyl-5-(4-(1H-Benzo[d]imidazole-2-Yl)phenyl)-1,3,4-Thiadiazole-2-Amine (5a)

Yield: 69%. Mp 325.6 °C. ^1^H NMR (300 MHz, DMSO-*d*_6_): δ = 3.25 (3H, s, −CH_3_), 7.98 (2H, d, J = 8.37 Hz, Aromatic CH), 8.11 (2H, s, d, J = 8.49 Hz, Aromatic CH), 8.25 (2H, d, J = 8.43 Hz, Aromatic CH), 8.30 (2H, d, J = 8.46 Hz, Aromatic CH), 13.08 (1H, s, NH). ^13^C NMR (75 MHz, DMSO-*d*_6_): δ 30.05, 122.52, 123.40, 126.62, 126.93, 127.38, 128.33, 130.41, 132.01, 132.67, 134.41, 135.47, 150.61, 150.89. HRMS (*m*/*z*): [M + H]^+^ calcd for C_16_H_13_N_5_S: 308.0964; found: 308.0946.

#### 4.1.7 *N*-Ethyl-5-(4-(1H-Benzo[d]imidazole-2-Yl)phenyl)-1,3,4-Thiadiazole-2-Amine (5b)

Yield: 66%. Mp 321.6 °C. ^1^H NMR (300 MHz, DMSO-*d*_6_): δ = 1.34–1.39 (3H, m, CH_3_), 4.09–4.14 (2H, m, CH_2_), 7.07–7.17 (2H, m, Aromatic CH), 7.24–7.28 (1H, m, Aromatic CH), 7.53–7.56 (1H, m, Aromatic CH), 7.64–7.69 (1H, m, Aromatic CH), 7.77–7.80 (1H, m, Aromatic CH), 7.94–7.99 (1H, m, Aromatic CH), 8.06–8.12 (1H, m, Aromatic CH). ^13^C NMR (75 MHz, DMSO-*d*_6_): δ (ppm) 20.79, 17.98, 103.18, 105.77, 109.72, 112.73, 117.41, 117.83, 124.16, 126.64, 128.84, 129.67, 134.35, 145.78, 150.76.

#### 4.1.8 *N*-(2-Chloroethyl)-5-(4-(1H-Benzo[d]imidazole-2-Yl)phenyl)-1,3,4-Thiadiazole-2-Amine (5c)

Yield: 67%. Mp 191.2 °C. ^1^H NMR (300 MHz, DMSO-*d*_6_): δ = 2.79–2.80 (4H, m, CH_2_), 7.65–7.71 (3H, m, Aromatic CH), 7.91–7.99 (3H, m, Aromatic CH), 8.33 (1H, d, J = 8.31 Hz, Aromatic CH), 8.60 (1H, d, J = 8.34 Hz, Aromatic CH).^13^C NMR (75 MHz, DMSO-*d*_6_): δ(ppm): 22.96, 45.82, 105.67, 109.51, 112.94, 114.71, 119.18, 121.67, 128.13, 129.10, 129.33, 130.15, 132.24, 148.68, 151.18.

#### 4.1.9 *N*-(2-Chlorophenyl)-5-(4-(1H-Benzo[d]imidazole-2-Yl)phenyl)-1,3,4-Thiadiazole-2-Amine (5d)

Yield: 74%. Mp 294.8 °C. ^1^H NMR (300 MHz, DMSO-*d*_6_): δ = 7.21–7.24 (4H, m, Aromatic C–H), 7.38–7.43 (2H, m, Aromatic C–H), 7.53 (1H, dd, J1 = 1.41 Hz, J2 = 7.95 Hz, Aromatic C–H), 8.03 (2H, d, J = 8.52 Hz, Aromatic C–H), 8.28–8.32 (3H, m, Aromatic C–H). ^13^C NMR (75 MHz, DMSO-*d*_6_): δ(ppm): 102.76, 107.44, 113.14, 114.29, 119.76, 121.19, 122.91, 124.68, 125.17, 126.28, 127.01, 127.74, 128.22, 129.88, 136.63, 141.83, 144.53, 150.35, 155.05. HRMS (*m*/*z*): [M + H]^+^ calcd for C_21_H_14_N_5_SCl: 404.0731; found: 404.0721.

#### 4.1.10 N-Cyclohexyl-5-(4-(1H-Benzo[d]imidazole-2-Yl)phenyl)-1,3,4-Thiadiazole-2-Amine (5e)

Yield: 74%. Mp 276.5 °C. ^1^H NMR (300 MHz, DMSO-*d*_6_): δ = 1.14–1.33 (8H, m, cyclohexyl CH), 1.71 (1H, s, cyclohexyl CH), 1.99–2.02 (2H, m, cyclohexyl CH), 7.47–7.49 (2H, m, Aromatic C–H), 7.54–7.58 (2H, m, Aromatic C–H), 7.91–7.94 (2H, m, Aromatic C–H), 8.24–8.27 (2H, m, Aromatic C–H), 11.37 (1H, s, NH), 13.04 (1H, s, NH). ^13^C NMR (75 MHz, DMSO-*d*_6_): δ (ppm) 24.64, 25.53, 32.29, 54.68, 114.71, 119.93, 124.23, 126.73, 127.69, 128.16, 129.24, 129.85, 132.32, 134.78, 148.52, 154.41, 168.61. HRMS (*m*/*z*): [M + H]^+^ calcd for C_21_H_21_N_5_S: 376.1590; found: 376.1581.

#### 4.1.11 *N*-Isopropyl-5-(4-(1H-Benzo[d]imidazole-2-Yl)phenyl)-1,3,4-Thiadiazole-2-Amine (5f)

Yield: 73%. Mp 324.7 °C. ^1^H NMR (300 MHz, DMSO-*d*_6_): δ = 1.23 (6H, d, J = 6.45 Hz, −CH_3_), 3.79–3.96 (1H, m, −CH), 7.22–7.25 (2H, m, Aromatic C–H), 7.61–7.63 (1H, m, Aromatic C–H), 7.93 (2H, d, J = 8.52 Hz, Aromatic C–H), 7.99 (1H, d, J= 7.17 Hz, Aromatic C–H), 8.26 (2H, d, J= 8.52 Hz, Aromatic C–H). ^13^C NMR (75 MHz, DMSO-*d*_6_): δ (ppm) 20.99, 25.14, 113.57, 116.89, 118.66, 124.99, 125.62, 126.66, 128.40, 129.15, 130.50, 132.92, 135.39, 148.17, 150.56.

#### 4.1.12 N-Propyl-5-(4-(1H-Benzo[d]imidazole-2-Yl)phenyl)-1,3,4-Thiadiazole-2-Amine (5g)

Yield: 70%. Mp 275.2 °C. ^1^H NMR (300 MHz, DMSO-*d*_6_): δ = 0.92–0.95 (3H, m, −CH_3_), 1.60–1.65 (2H, m, −CH), 3.33 (2H, s, CH_2_), 7.59–7.61 (2H, m, Aromatic C–H), 7.88–7.90 (2H, m, Aromatic C–H), 8.12–8.15 (2H, m, Aromatic C–H), 8.28–8.32 (2H, m, Aromatic C–H). ^13^C NMR (75 MHz, DMSO-*d*_6_): δ (ppm) 12.68, 23.69, 25.46, 112.63, 117.51, 121.57, 121.98, 123.85, 125.92, 127.28, 128.53, 129.31, 130.71, 138.29, 152.22, 154.82. HRMS (*m*/*z*): [M + H]^+^ calcd for C_18_H_17_N_5_S: 336.1277; found: 336.1271.

#### 4.1.13 *N*-(4-Methoxyphenyl)-5-(4-(1H-Benzo[d]imidazole-2-Yl)phenyl)-1,3,4-Thiadiazole-2-Amine (5h)

Yield: 72%. Mp 276.7 °C. ^1^H NMR (300 MHz, DMSO-*d*_6_): δ = 3.75 (3H, s, -OCH_3_), 6.95–6.99 (2H, m, Aromatic C–H), 7.22–7.24 (2H, m, Aromatic C–H), 7.57 (2H, d, J = 9.00 Hz, Aromatic C–H), 7.68–7.77 (2H, m, Aromatic C–H), 8.02 (2H, d, J = 8.46 Hz, Aromatic C–H), 8.29 (2H, d, J = 8.46 Hz, Aromatic C–H). ^13^C NMR (75 MHz, DMSO-*d*_6_): δ (ppm) 55.69, 113.14, 114.82, 115.60, 120.00, 123.31, 123.41, 127.08, 127.64, 127.81, 128.58, 130.67, 132.29, 134.39, 150.43, 155.25, 156.54, 165.60. HRMS (*m*/*z*): [M + H]^+^ calcd for C_22_H_17_N_5_OS: 400.1227; found: 400.1228.

#### 4.1.14 N-Isobutyl-5-(4-(1H-Benzo[d]imidazole-2-Yl)phenyl)-1,3,4-Thiadiazole-2-Amine (5i)

Yield: 70%. Mp 315.6 °C. ^1^H NMR (300 MHz, DMSO-*d*_6_): δ = 0.94 (6H, d, J = 6.66 Hz, CH_3_), 1.89–1.97 (1H, m, CH), 3.17 (2H, m, CH_2_), 7.23–7.24 (2H, m, Aromatic CH), 7.67–7.69 (1H, m, Aromatic CH), 7.92 (2H, d, 8.46 Hz, Aromatic CH), 8.10–8.14 (1H, m, Aromatic CH), 8.25 (2H, d, 8.46 Hz, Aromatic CH). ^13^C NMR (75 MHz, DMSO-*d*_6_): δ (ppm) 19.85, 28.78, 61.20, 103.49, 108.06, 112.22, 114.40, 121.25, 124.58, 126.49, 131.13, 134.76, 135.28, 148.89, 150.87, 161.78. HRMS (*m*/*z*): [M + H]^+^ calcd for C_19_H_19_N_5_S: 350.1434; found: 350.1421.

### 4.2 Antimicrobial Activity

The antimicrobial activity of the final compounds (**5a**–**i**) was screened against eight species of pathogenic bacteria and three fungal species (*E. coli* (ATCC 25922), *S. marcescens* (ATCC 8100), *K. pneumonia* (ATCC 13883), *P. aeruginosa* (ATCC 27853), *E. faecalis* (ATCC 2942), *B. subtilis* (NRRL NRS 744), *S. aureus* (ATCC 29213), *S. epidermidis* (ATCC 12228), *C. albicans* (ATCC 24433), *C. glabrata* (ATCC 90030), *C. krusei* (ATCC 6258), and *C. parapsilosis* (ATCC 22 019)) according to the microdilution standard methods CLSI M07-A9 (2012) and NCCLS M27-A2 (2002), as described in the previous study.^25,26^

### 4.3 Cytotoxicity Assay

The effect of the compounds between **5a**–**i** on the viability of the L929 cell line was analyzed by MTT assay. The MTT method was performed as previously described.^27^

### 4.4 *In Silico* Studies

#### 4.4.1 Quantum Chemical Calculations

Using the gradient-corrected correlation functional of Lee, Yang, and Parr (LYP) and Becke’s three- parameter exchange functional (B3), electronic characterization of the produced compounds was performed in the gas phase. The optimization was validated using frequency calculations, and the structures’ minimum energy was found when imaginary frequencies were absent.^28^ The same technique was used to perform MESP analysis using these optimized structure HOMO–LUMO energies (electronic characteristics, such as the ionization potential, electronegativity, electrophilic index, nucleophilic index, and chemical potential produced from these energies). Quantum computations were carried out using Jaguar software, and the molecular orbitals were examined using the maestro interface.

#### 4.4.2 Molecular Docking and Molecular Dynamic (MD) Simulation Studies

The Schrödinger Glide was used to conduct molecular docking studies for the newly thiadiazole derivatives (**5a**–**i**) on the crystal structure of sterol 14-α demethylase (CYP51) from *C. albicans* cocrystallized with the tetrazole-based antifungal drug candidate VT1161(PDB ID: 5TZ1) receptor to evaluate their *insilico* inhibitory effects. The protein preparation wizard was used to protonate the protein under physiological pH and to apply the OPLS3e force field to these two sensors.^29,30^ As previously mentioned, each protein was rectified and 3D hydrogenated, and energy was minimized. Chem. Draw was used to create the 2D structures of the pyrimidine derivatives (**5a**–**i**). Then, LigPrep was used to generate 3D structures, add charges, minimize energy, and compile all structures into a single molecular database file.^31^ Finally, docking investigations were carried out utilizing the Glide docking wizard and Standard Procedure (SP) as the docking procedure, with docking results visualized using the Maestro Graphical User Interface (GUI). To determine the atomic-level binding stability of the top-ranked compounds and gain insights into their molecular interactions, molecular dynamics (MD) simulations were conducted using the Desmond module of Schrödinger. The 5f–5TZ1 complex and 5h–5TZ1 complexes were solvated under orthorhombic periodic boundary conditions, maintaining a 10 Å buffer region between protein atoms and box edges with the explicit SPC water model. In the system builder, sodium and chloride ions were introduced to neutralize charges, and a 0.15 M NaCl salt concentration was added to mimic human physiological conditions.^32^ The built system was then minimized using the fixed parameters of the OPLS3e force field to eliminate electronic clashes and appropriately align the protein structure within the simulation boundaries.^33^ Long-range electrostatic interactions were evaluated using the smooth particle mesh Ewald approach with a tolerance of 1e–09, while short-range van der Waals and Coulomb interactions were computed with a cutoff radius of 9.0 Å. After importing the minimized build system (.cms file) into the molecular dynamics module, a 100 ns simulation was conducted under an’isothermal–isobaric ensemble’ (NPT) at a temperature of 300 K and a pressure of 1 bar. The’Nose-Hoover chain thermostat’ and’Martyna-Tobias-Klein barostat’ techniques were employed at 100 and 200 ps intervals for isothermal–isobaric conditions, respectively.^34^ Simulation snapshots were retrieved at 100 ps intervals, and the resulting trajectories were analyzed. All computational modeling is performed on a workstation, featuring Ubuntu 22.04.2 LTS 64-bit configuration, Intel Xeon W-2245 @ 3.90 GHz, 8 cores, CUDA 12, and NVIDIA RTX A4000 graphics processing unit.

## Supporting Information Available

^1^H NMR, ^13^C NMR, and HRMS spectra of compounds **5a**–**5i** (PDF)
