# Supplementary material for: Synthesis, DFT Calculations, In Silico Studies, and Antimicrobial Evaluation of Benzimidazole-Thiadiazole Derivatives
Source: ACS Omega. 2024 Apr 9;9(16):18469–79. doi: 10.1021/acsomega.4c00543 (PMC11044166; doi:10.1021/acsomega.4c00543)
Supplement: Supplementary file 1 — ao4c00543_si_001.pdf [file ao4c00543_si_001.pdf]

Synthesis, DFT calculations, *in silico* studies, and antimicrobial evaluation of benzimidazole-thiadiazole derivatives

Ayşen Işık[1], Ulviye Acar Çevik[2*], Arzu Karayel[3], Iqrar Ahmad[4,5], Harun Patel[5], İsmail Çelik[6], Ülküye Dudu Gül[7], Gizem Bayazıt[8], Hayrani Eren Bostancı[9], Ahmet Koçak[10], Yusuf Özkay[2], Zafer Asım Kaplancıklı[2]

[1] Department of Biochemistry, Faculty of Science, Selçuk University, Konya, Turkey.

[2] Department of Pharmaceutical Chemistry, Faculty of Pharmacy, Anadolu University, Eskişehir 26470, Turkey.

[3] Department of Physics, Faculty of Arts and Science, Hitit University, 19030 Çorum, Turkey.

[4] Department of Pharmaceutical Chemistry, Prof. Ravindra Nikam College of Pharmacy, Gondur, Dhule, 424002, Maharashtra, India

[5] Division of Computer Aided Drug Design, Department of Pharmaceutical Chemistry, R. C. Patel Institute of Pharmaceutical Education and Research, Shirpur,425405, Maharashtra, India

[6] Department of Pharmaceutical Chemistry, Faculty of Pharmacy, Erciyes University, Kayseri 38039, Turkey.

[7] Department of Bioengineering, Faculty of Engineering, Bilecik Seyh Edebali University, Bilecik, Turkey

[8] Department of Biotechnology, Institute of Graduate Studies, Bilecik Seyh Edebali University, Bilecik, Turkey

[9] Department of Biochemistry, Faculty of Pharmacy, Cumhuriyet University, Sivas, Turkey

[10] Department of Chemistry, Faculty of Science, Selçuk University, Konya, Turkey.

* Corresponding Author. E-mail: uacar@anadolu.edu.tr; Tel. +90-222-335-0580/3775

Address: Anadolu University, Faculty of Pharmacy, Department of Pharmaceutical Chemistry, 26470, Eskişehir, Turkey.

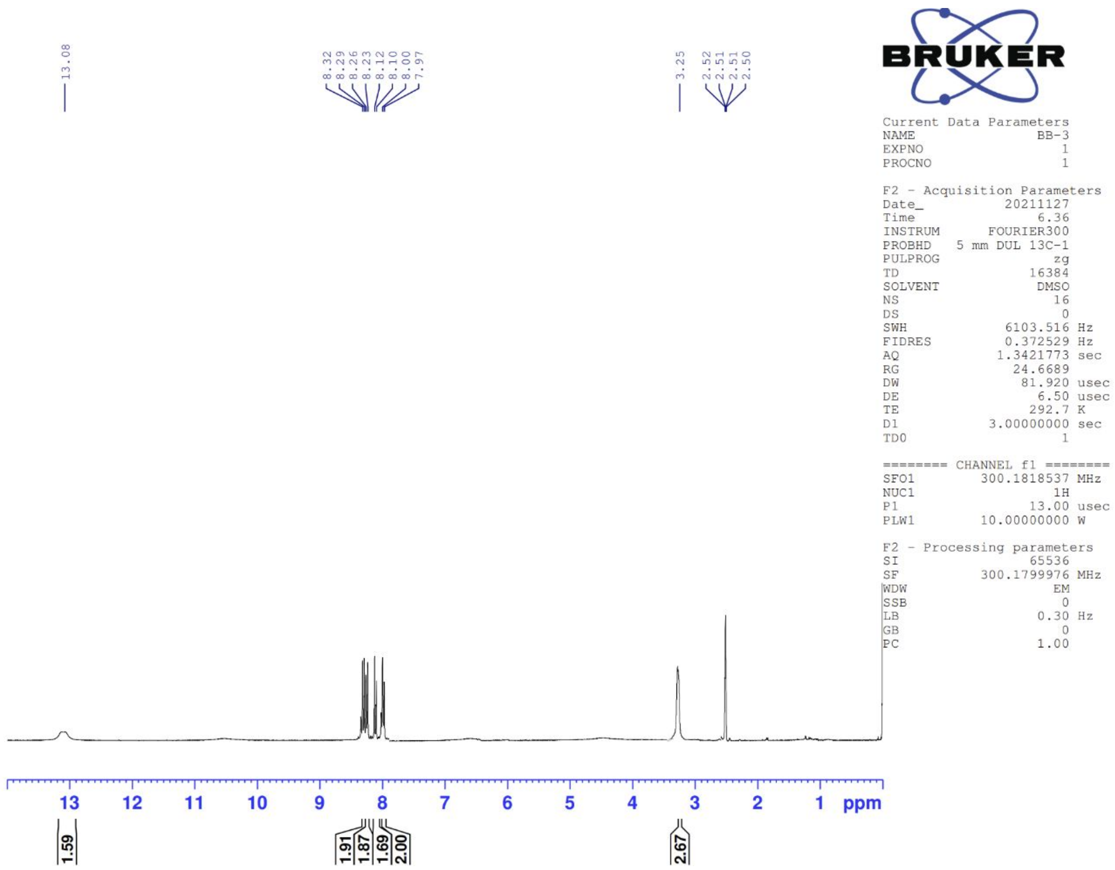

Figure S1. ^{1}H-NMR spectrum of compound **5a**

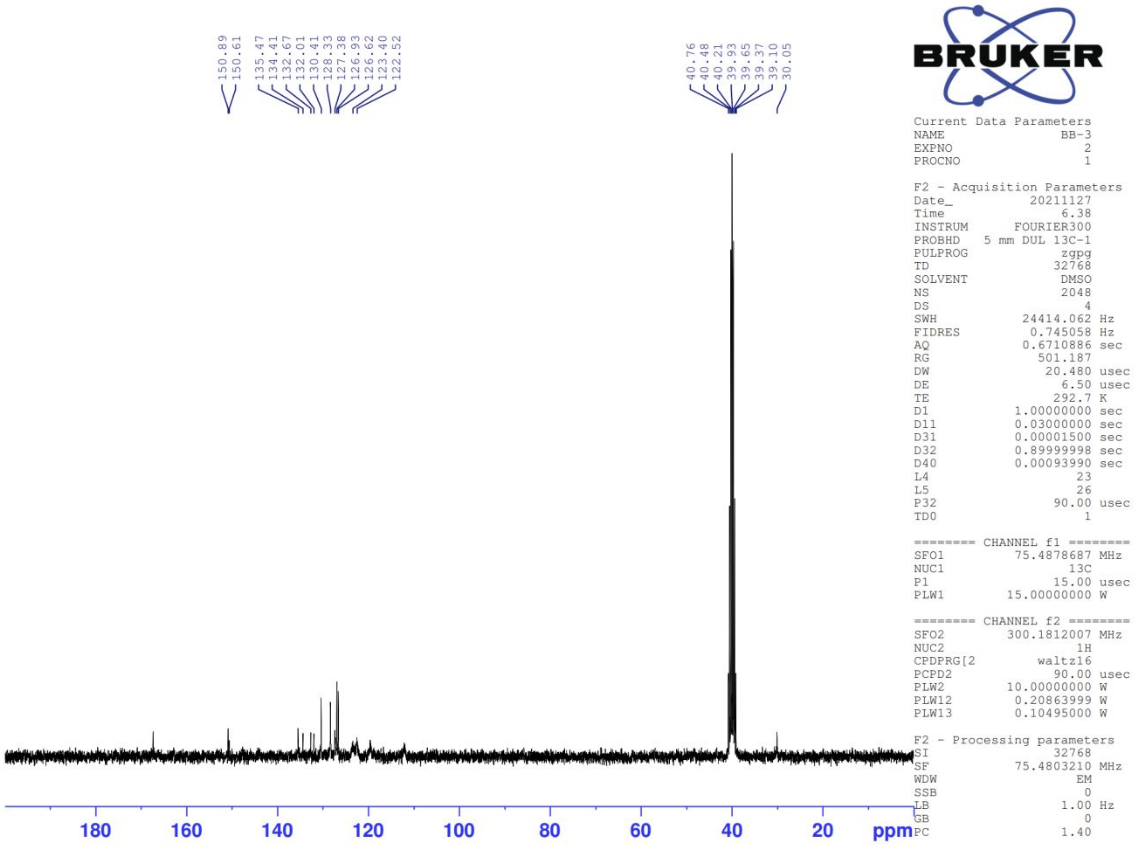

Figure S2. ^{13}C-NMR spectrum of compound **5a**

Data File: C:\LabSolutions\Data\Analiz\uac\BB-1_58.lcd

Elmt	Val.	Min	Max	Elmt	Val.	Min	Max	Elmt	Val.	Min	Max	Elmt	Val.	Min	Max	Use Adduct
H	1	10	40	O	2	0	4	S	2	1	1	Ru	2	0	0	H
C	4	9	40	F	1	0	0	Cl	1	0	0	Pd	2	0	0	
N	3	2	8	P	3	0	0	Br	1	0	0	I	3	0	0	

Error Margin (ppm):	7	DBE Range:	5.0 - 25.0	Electron Ions:	both
HC Ratio:	unlimited	Apply N Rule:	yes	Use MSn Info:	yes
Max Isotopes:	3	Isotope RI (%):	1.00	Isotope Res:	9000
MSn Iso RI (%):	10.00	MSn Logic Mode:	AND	Max Results:	150

Event#: 1 MS(E+) Ret. Time : 3.053 Scan# : 459

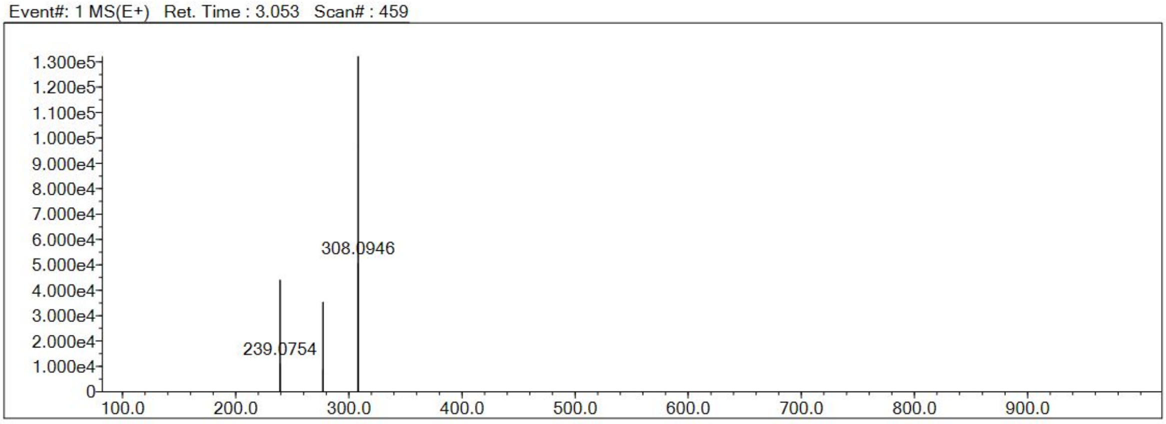

Measured region for 308.0946 m/z

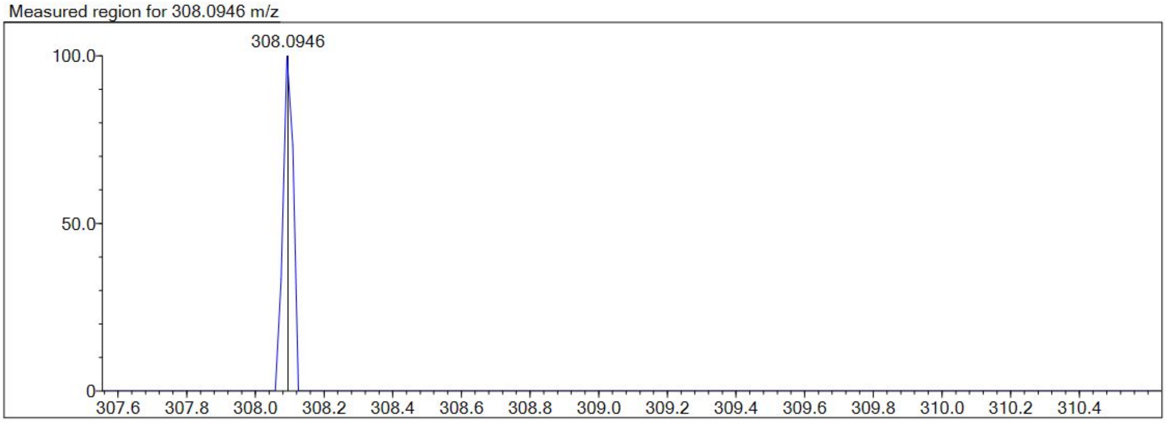

C16 H13 N5 S [M+H]+ : Predicted region for 308.0964 m/z

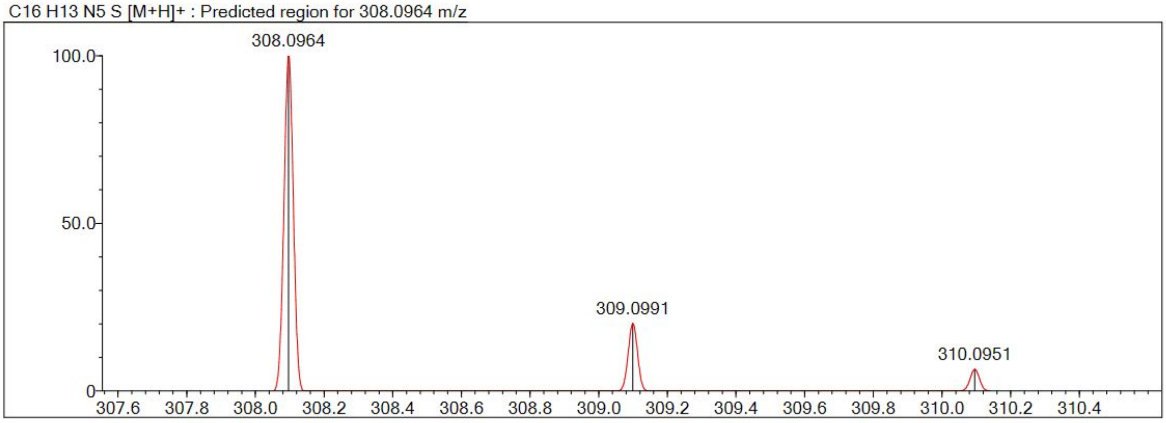

Rank	Score	Formula (M)	Ion	Meas. m/z	Pred. m/z	Df. (mDa)	Df. (ppm)	Iso	DBE
1	0.00	C16 H13 N5 S	[M+H]+	308.0946	308.0964	-1.8	-5.84	0.00	13.0

Figure S3. Mass spectrum of compound **5a**

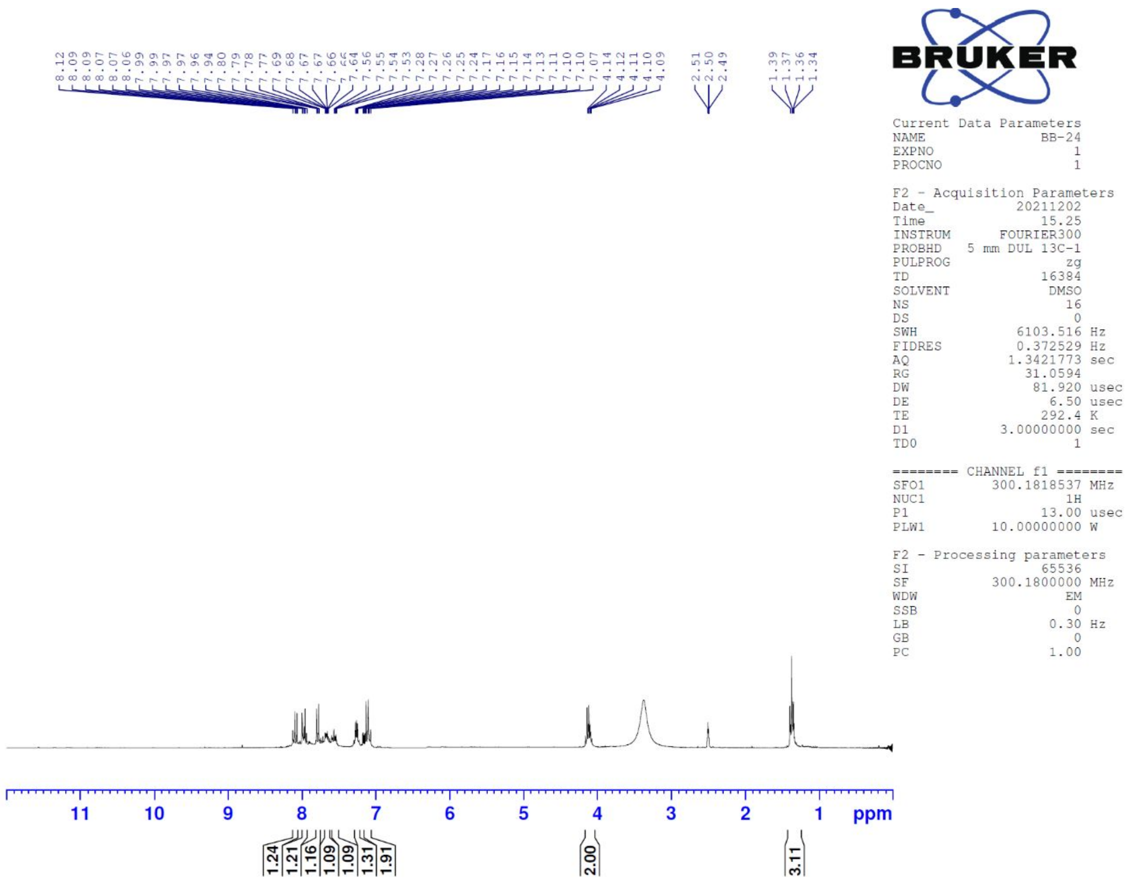

Figure S4. ^{1}H-NMR spectrum of compound **5b**

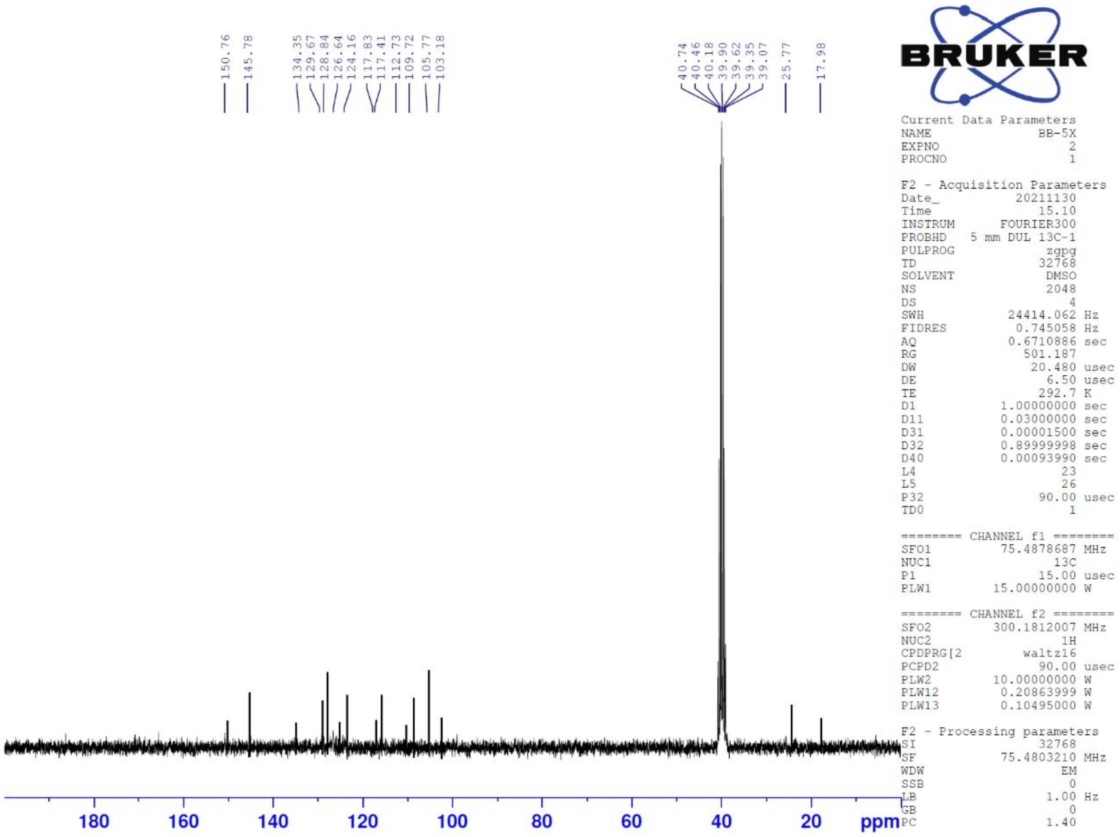

Figure S5. ^{13}C-NMR spectrum of compound **5b**

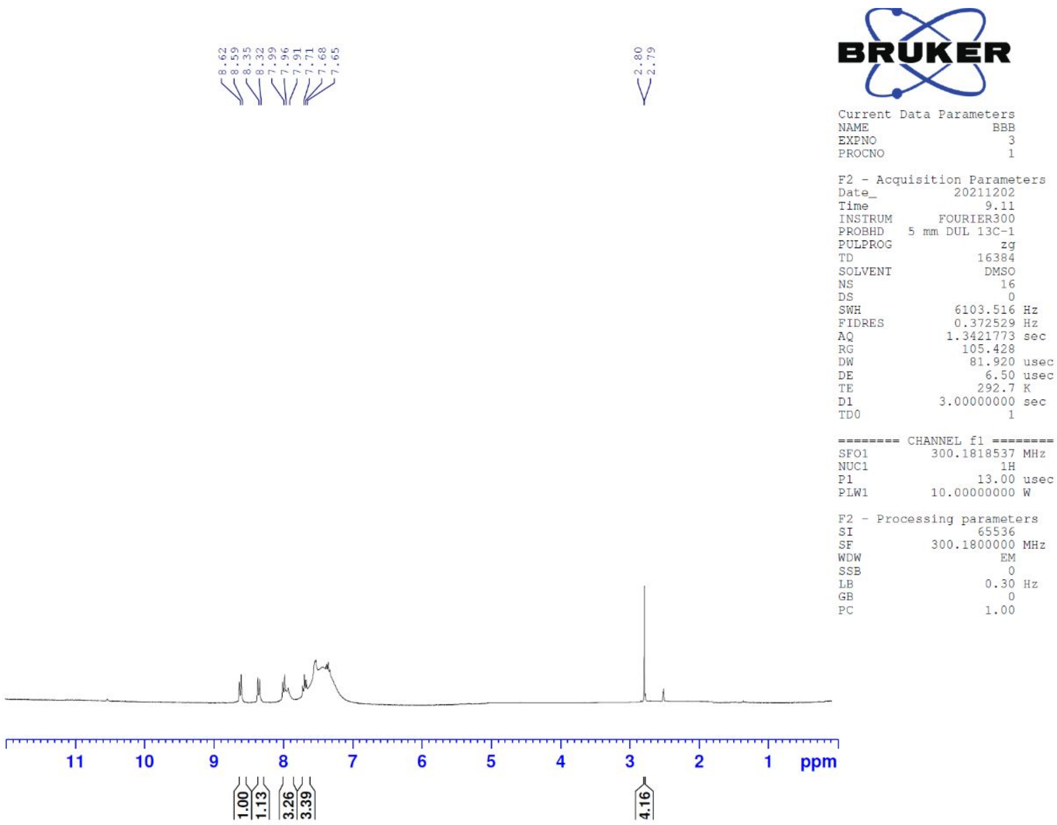

Figure S6. ^{1}H-NMR spectrum of compound **5c**

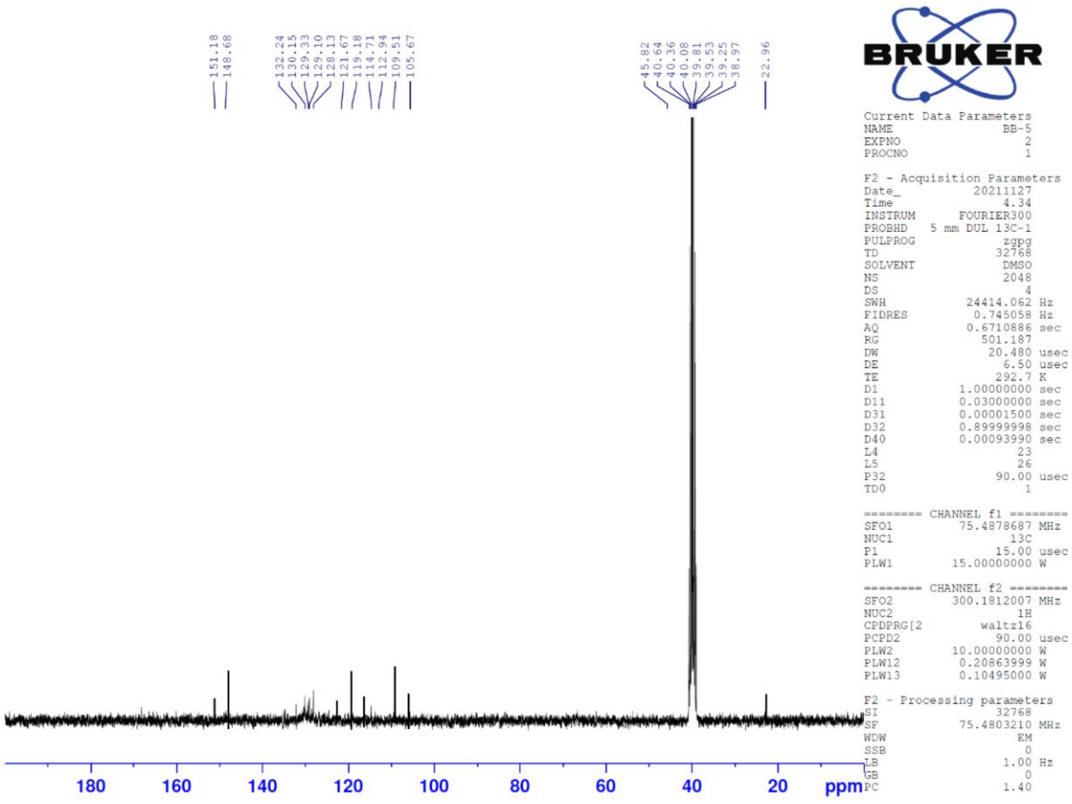

Figure S7. ^{13}C-NMR spectrum of compound **5c**

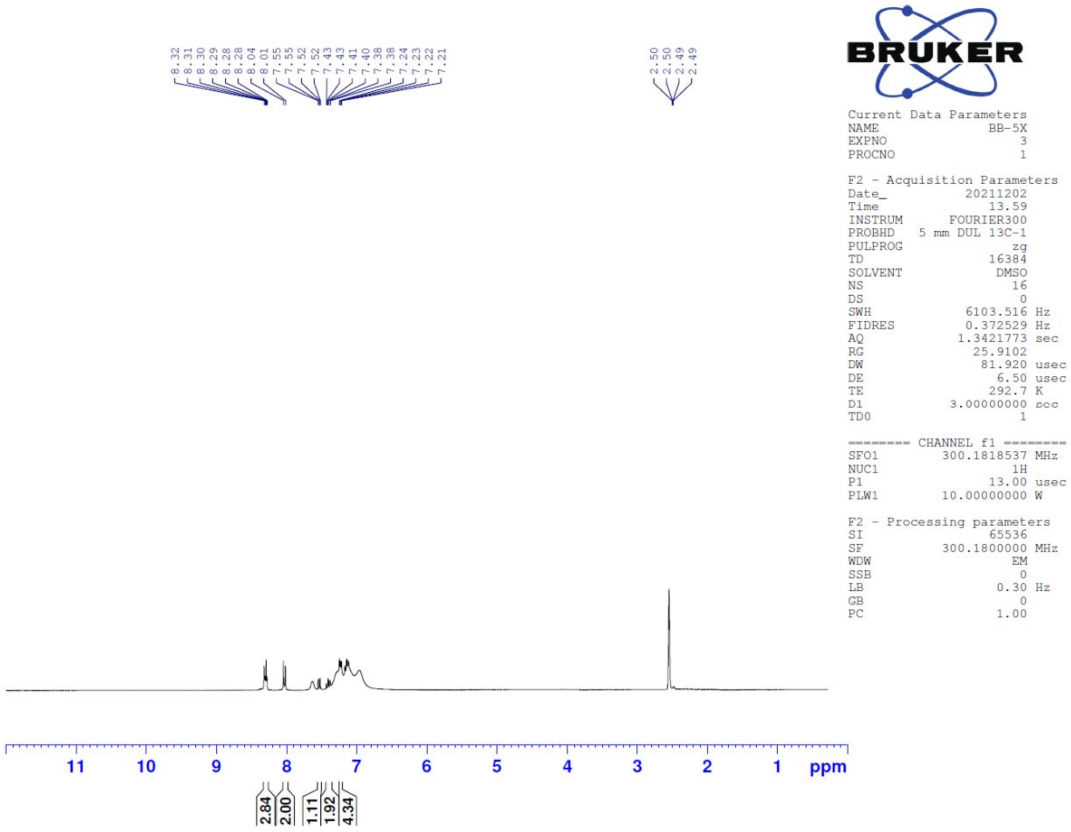

Figure S8. ^{1}H-NMR spectrum of compound **5d**

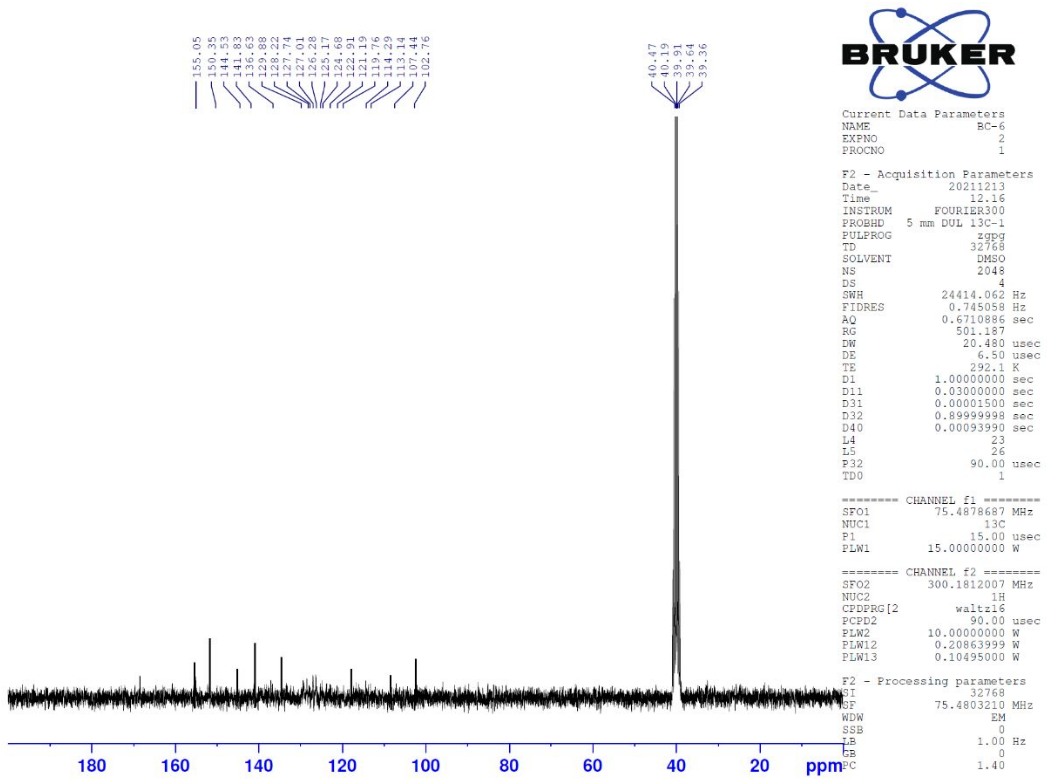

Figure S9. ^{13}C-NMR spectrum of compound **5d**

Data File: C:\LabSolutions\Data\Analiz\uac\BB-5_60.lcd

Elmt	Val.	Min	Max	Elmt	Val.	Min	Max	Elmt	Val.	Min	Max	Elmt	Val.	Min	Max	Use Adduct
H	1	10	40	O	2	0	4	S	2	1	1	Ru	2	0	0	H
C	4	9	40	F	1	0	0	Cl	1	1	1	Pd	2	0	0	
N	3	2	8	P	3	0	0	Br	1	0	0	I	3	0	0	

Error Margin (ppm):	7	DBE Range:	5.0 - 25.0	Electron Ions:	both
HC Ratio:	unlimited	Apply N Rule:	yes	Use MSn Info:	yes
Max Isotopes:	3	Isotope RI (%):	1.00	Isotope Res:	9000
MSn Iso RI (%):	10.00	MSn Logic Mode:	AND	Max Results:	150

Event#: 1 MS(E+) Ret. Time : 4.013 Scan# : 603

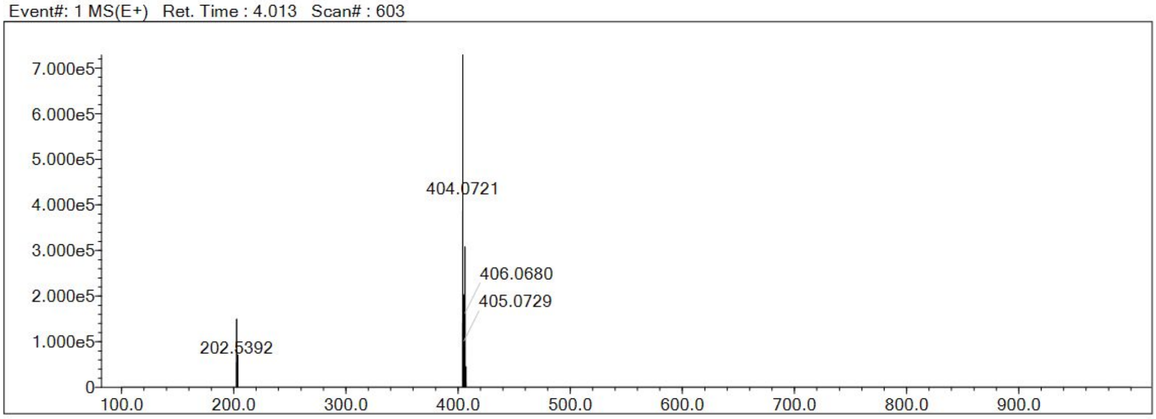

Measured region for 404.0721 m/z

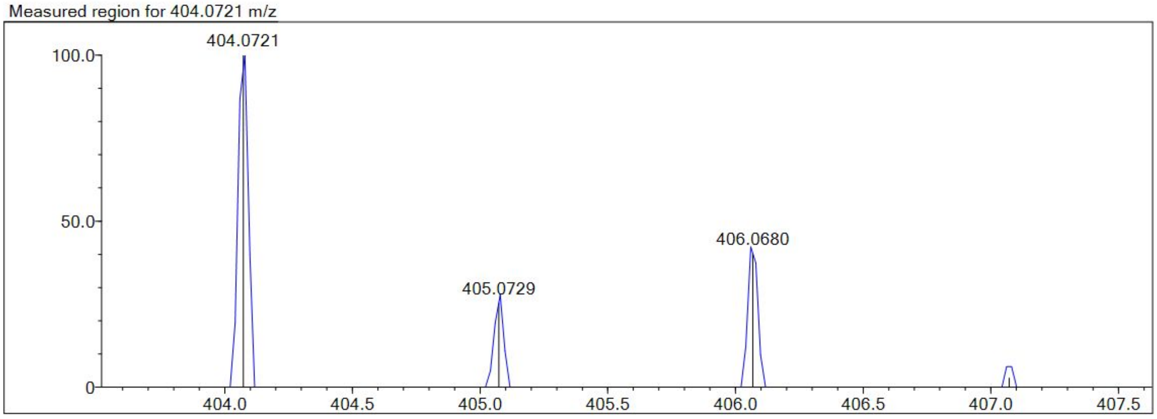

C21 H14 N5 S Cl [M+H]+ : Predicted region for 404.0731 m/z

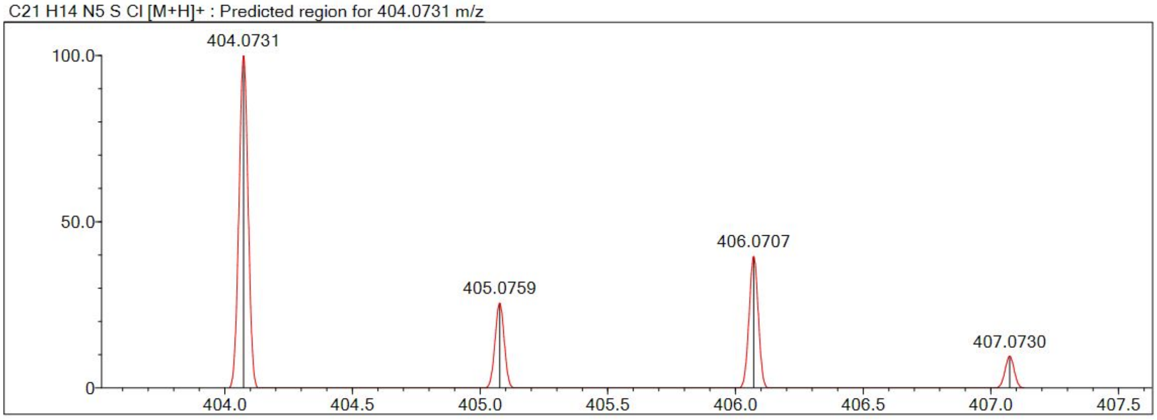

Rank	Score	Formula (M)	Ion	Meas. m/z	Pred. m/z	Df. (mDa)	Df. (ppm)	Iso	DBE
1	81.02	C21 H14 N5 S Cl	[M+H]+	404.0721	404.0731	-1.0	-2.47	84.11	17.0

Figure S10. Mass spectrum of compound **5d**

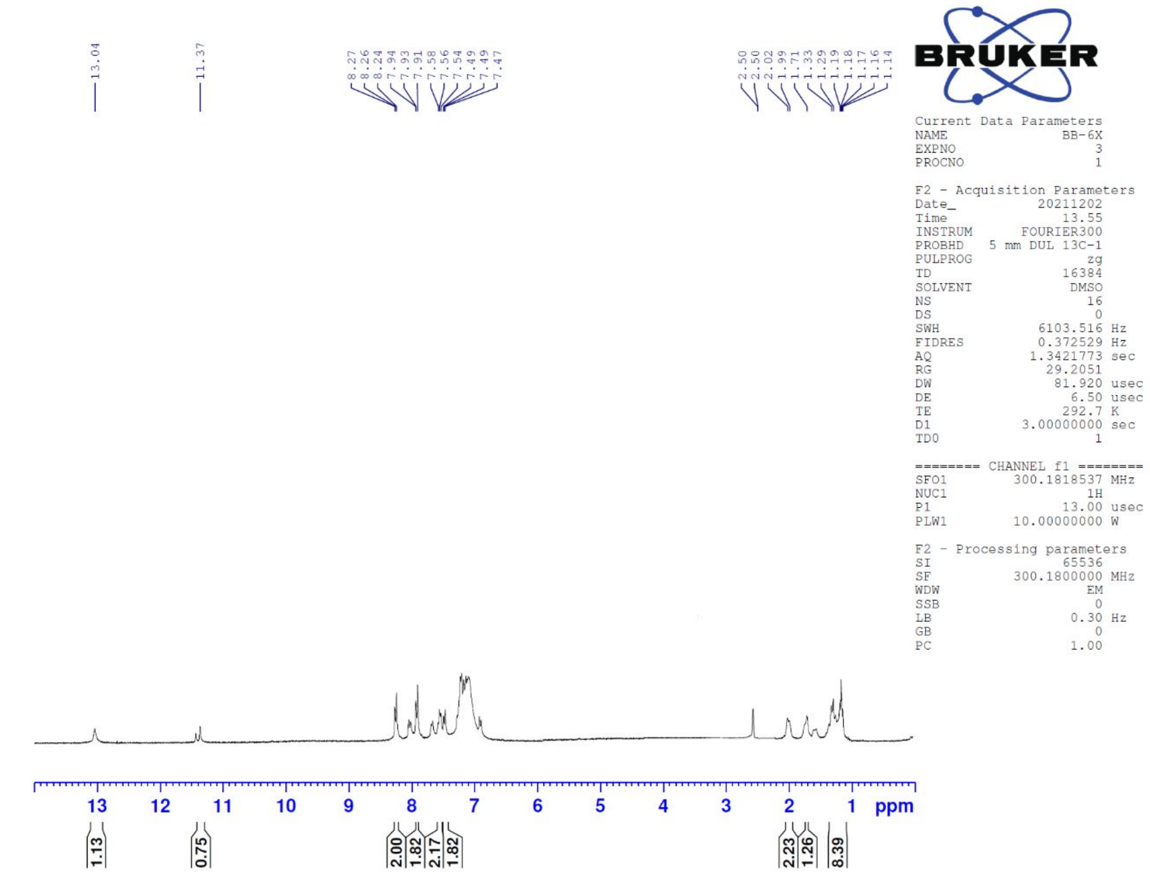

Figure S11. ^{1}H-NMR spectrum of compound **5e**

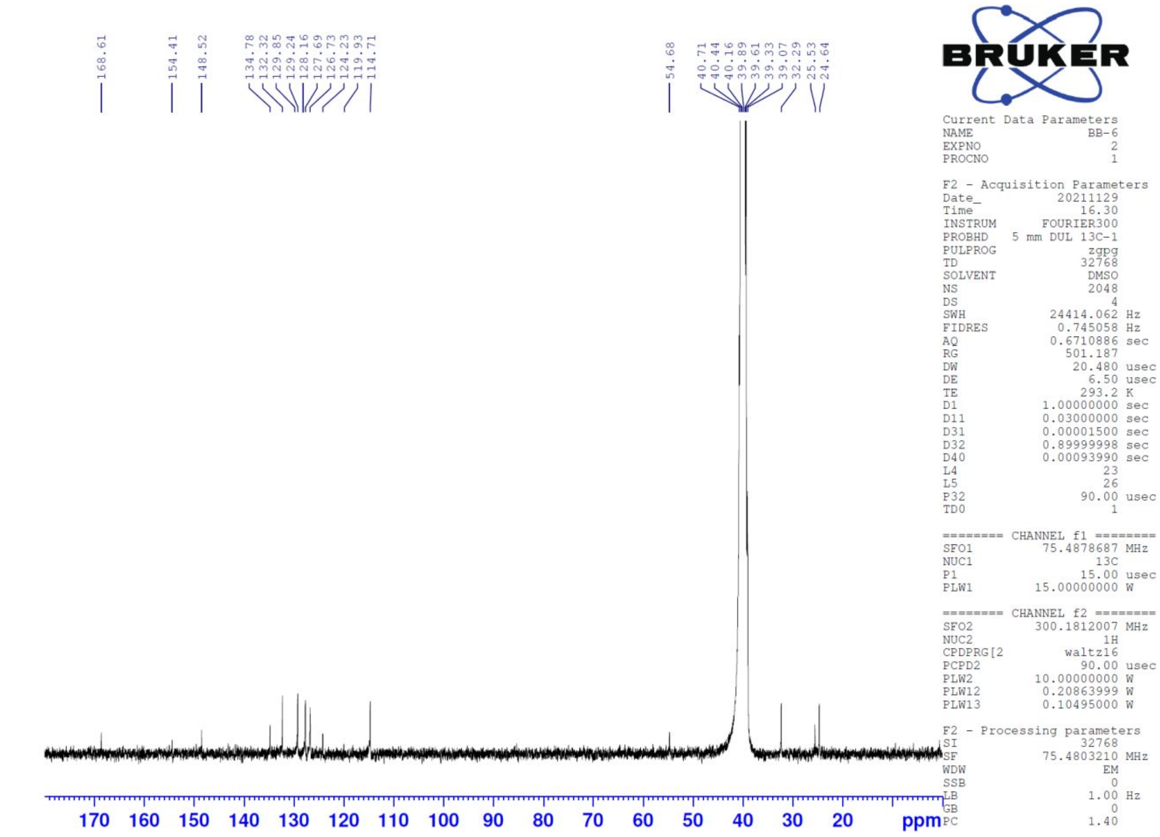

Figure S12. ^{13}C-NMR spectrum of compound **5e**

Data File: C:\LabSolutions\Data\Analiz\uac\BB-6_62.lcd

Elmt	Val.	Min	Max	Elmt	Val.	Min	Max	Elmt	Val.	Min	Max	Elmt	Val.	Min	Max	Use Adduct
H	1	10	40	O	2	0	4	S	2	1	1	Ru	2	0	0	H
C	4	9	40	F	1	0	0	Cl	1	0	0	Pd	2	0	0	
N	3	2	8	P	3	0	0	Br	1	0	0	I	3	0	0	

Error Margin (ppm):	7	DBE Range:	5.0 - 25.0	Electron Ions:	both
HC Ratio:	unlimited	Apply N Rule:	yes	Use MSn Info:	yes
Max Isotopes:	3	Isotope RI (%):	1.00	Isotope Res:	9000
MSn Iso RI (%):	10.00	MSn Logic Mode:	AND	Max Results:	150

Event#: 1 MS(E+) Ret. Time : 3.933 Scan# : 591

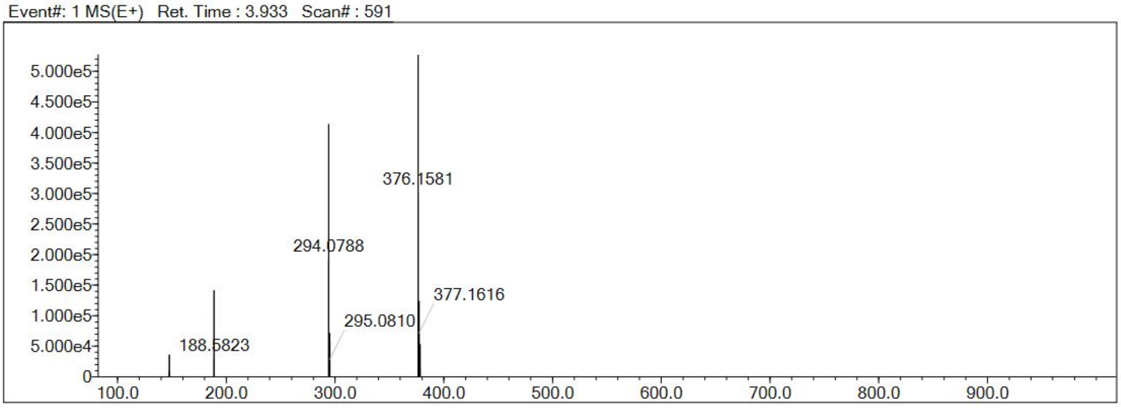

Measured region for 376.1581 m/z

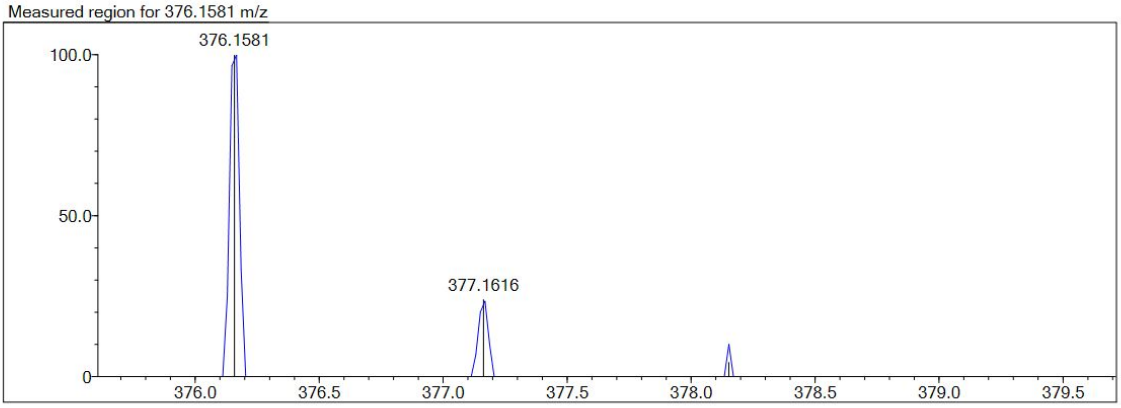

C21 H21 N5 S [M+H]+ : Predicted region for 376.1590 m/z

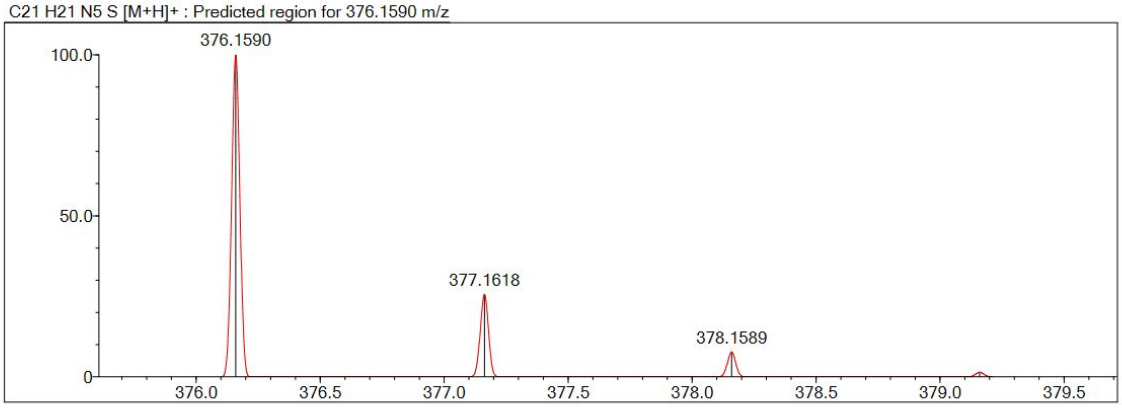

Rank	Score	Formula (M)	Ion	Meas. m/z	Pred. m/z	Df. (mDa)	Df. (ppm)	Iso	DBE
1	75.06	C21 H21 N5 S	[M+H]+	376.1581	376.1590	-0.9	-2.39	77.76	14.0

Figure S13. ^{1}H-NMR spectrum of compound **5e**

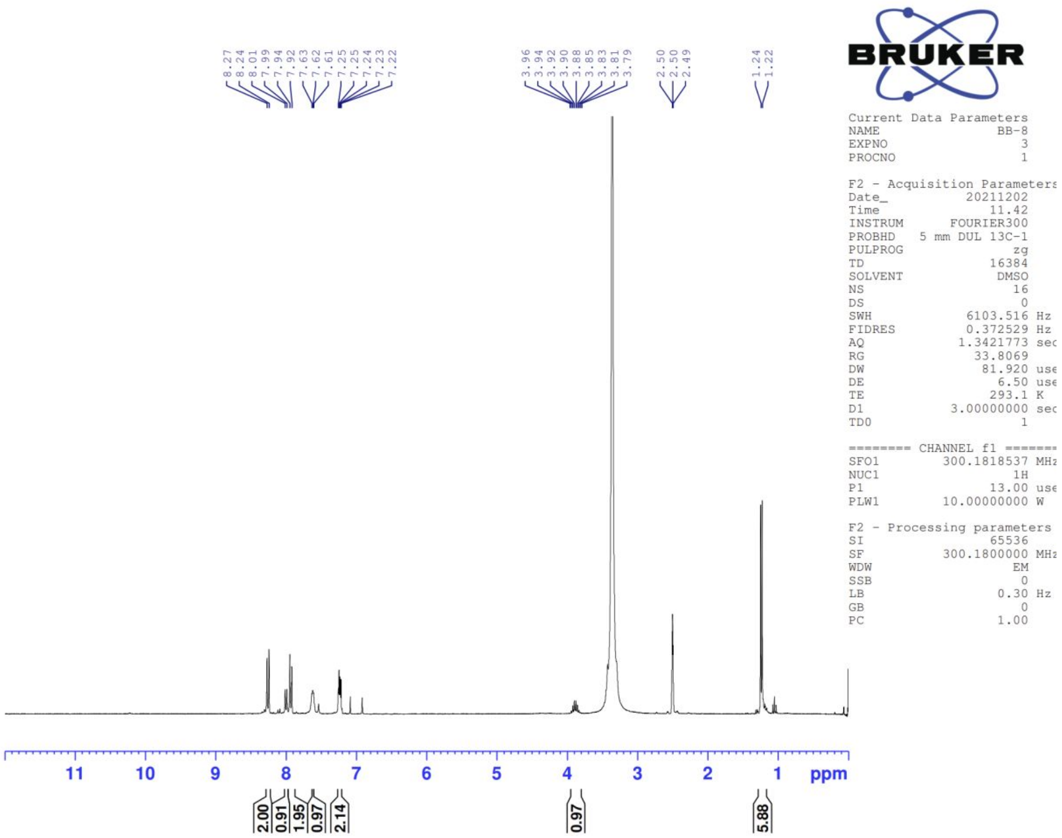

Figure S14. ^{1}H-NMR spectrum of compound **5f**

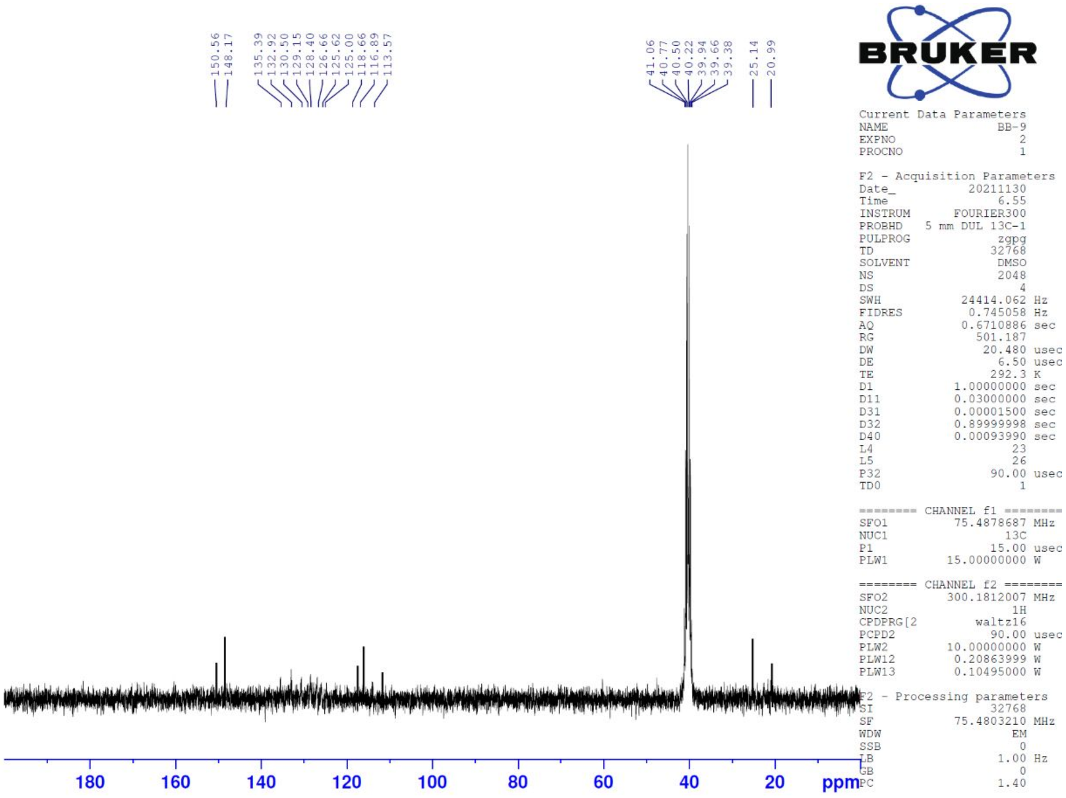

Figure S15. ^{13}C-NMR spectrum of compound **5f**

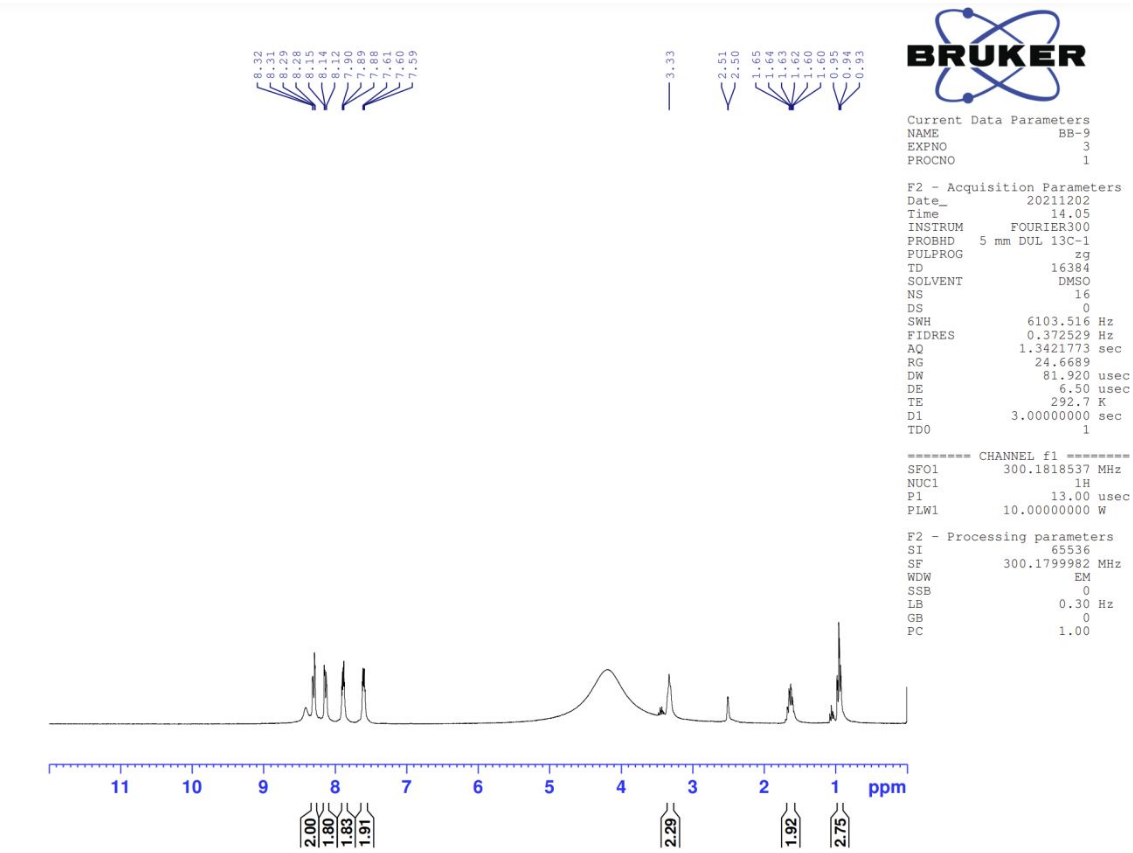

Figure S16. ^{1}H-NMR spectrum of compound **5g**

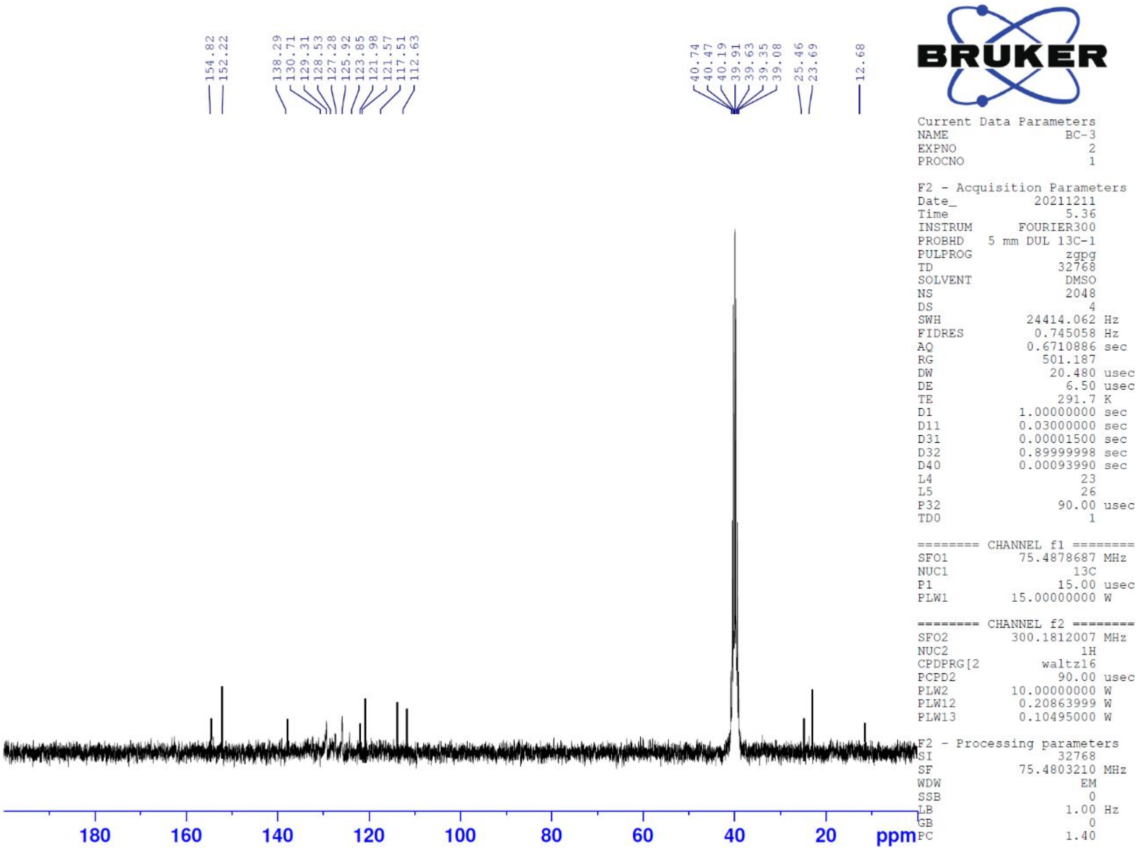

Figure S17. ^{13}C-NMR spectrum of compound **5g**

Data File: C:\LabSolutions\Data\Analiz\uac\BB-9_66.lcd

Elmt	Val.	Min	Max	Elmt	Val.	Min	Max	Elmt	Val.	Min	Max	Elmt	Val.	Min	Max	Use Adduct
H	1	10	40	O	2	0	4	S	2	1	1	Ru	2	0	0	H
C	4	9	40	F	1	0	0	Cl	1	0	0	Pd	2	0	0	
N	3	2	8	P	3	0	0	Br	1	0	0	I	3	0	0	

Error Margin (ppm): 7
HC Ratio: unlimited
Max Isotopes: 3
MSn Iso RI (%): 10.00
DBE Range: 5.0 - 25.0
Apply N Rule: yes
Isotope RI (%): 1.00
MSn Logic Mode: AND
Electron Ions: both
Use MSn Info: yes
Isotope Res: 9000
Max Results: 150

Event#: 1 MS(E+) Ret. Time : 3.253 Scan# : 489

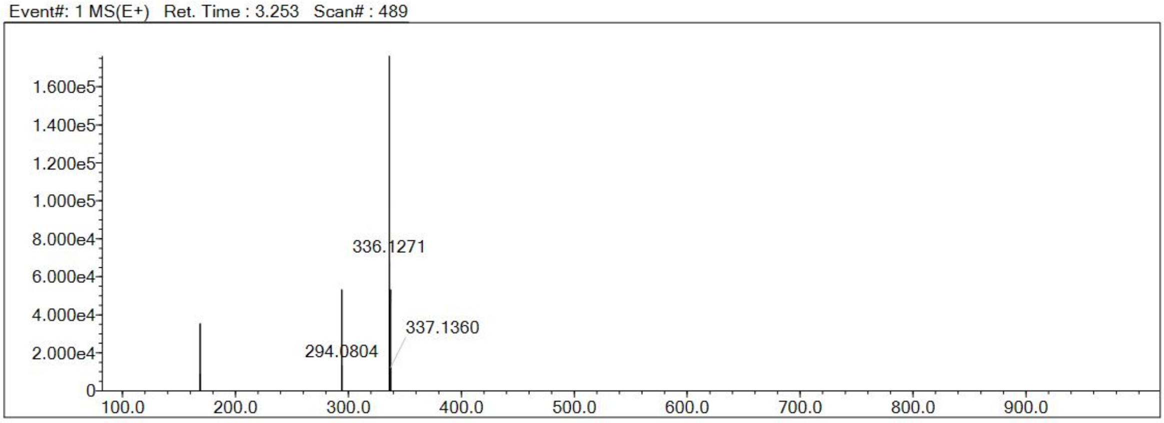

Measured region for 336.1271 m/z

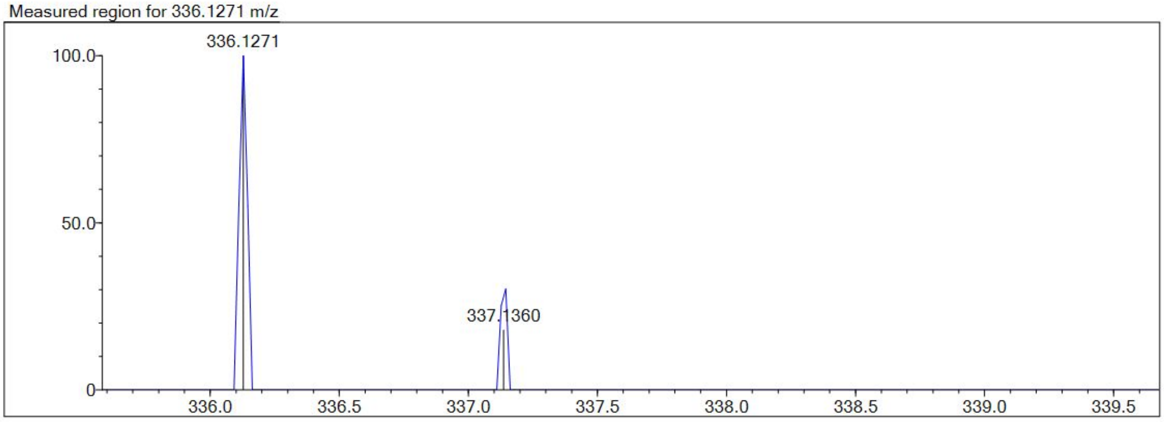

C18 H17 N5 S [M+H]+ : Predicted region for 336.1277 m/z

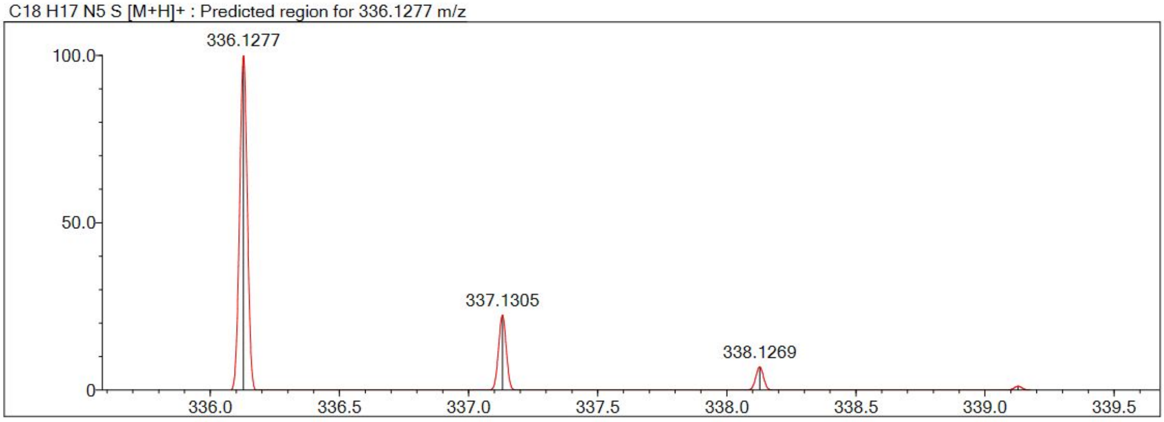

Rank	Score	Formula (M)	Ion	Meas. m/z	Pred. m/z	Df. (mDa)	Df. (ppm)	Iso	DBE
1	0.00	C18 H17 N5 S	[M+H]+	336.1271	336.1277	-0.6	-1.79	0.00	13.0

Figure S18. Mass spectrum of compound **5g**

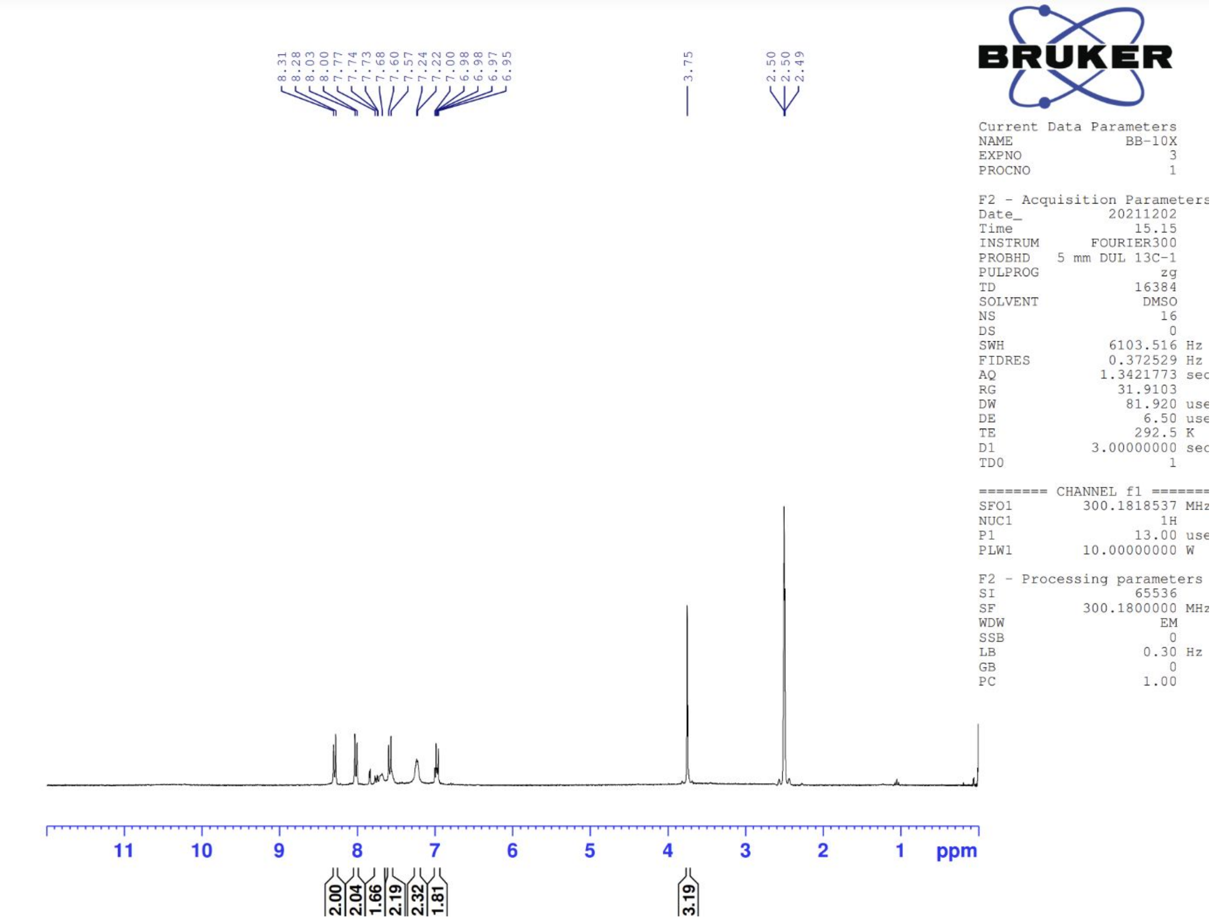

Figure S19. ^{1}H-NMR spectrum of compound **5h**

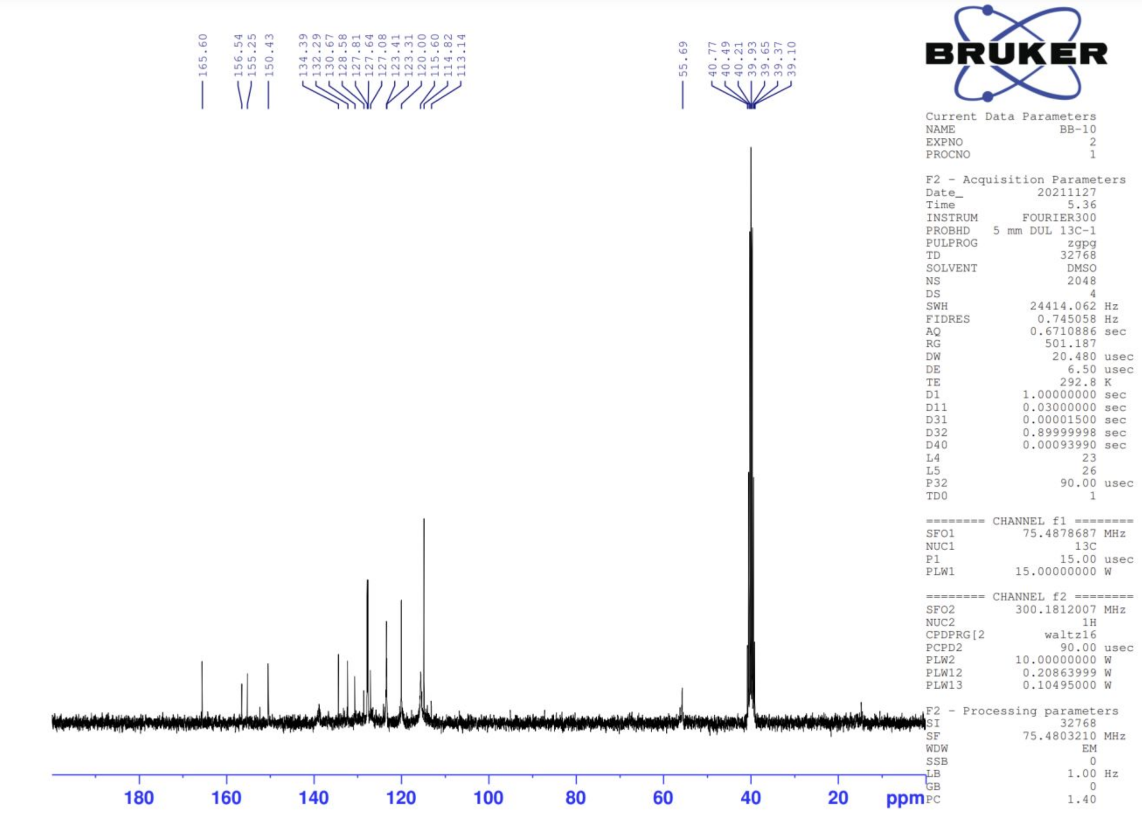

Figure S20. ^{13}C-NMR spectrum of compound **5h**

Data File: C:\LabSolutions\Data\Analiz\uac\BB-10_67.lcd

Elmt	Val.	Min	Max	Elmt	Val.	Min	Max	Elmt	Val.	Min	Max	Elmt	Val.	Min	Max	Use Adduct
H	1	10	40	O	2	0	4	S	2	1	1	Ru	2	0	0	H
C	4	9	40	F	1	0	0	Cl	1	0	0	Pd	2	0	0	
N	3	2	8	P	3	0	0	Br	1	0	0	I	3	0	0	

Error Margin (ppm):	7	DBE Range:	5.0 - 25.0	Electron Ions:	both
HC Ratio:	unlimited	Apply N Rule:	yes	Use MSn Info:	yes
Max Isotopes:	3	Isotope RI (%):	1.00	Isotope Res:	9000
MSn Iso RI (%):	10.00	MSn Logic Mode:	AND	Max Results:	150

Event#: 1 MS(E+) Ret. Time : 3.360 Scan# : 505

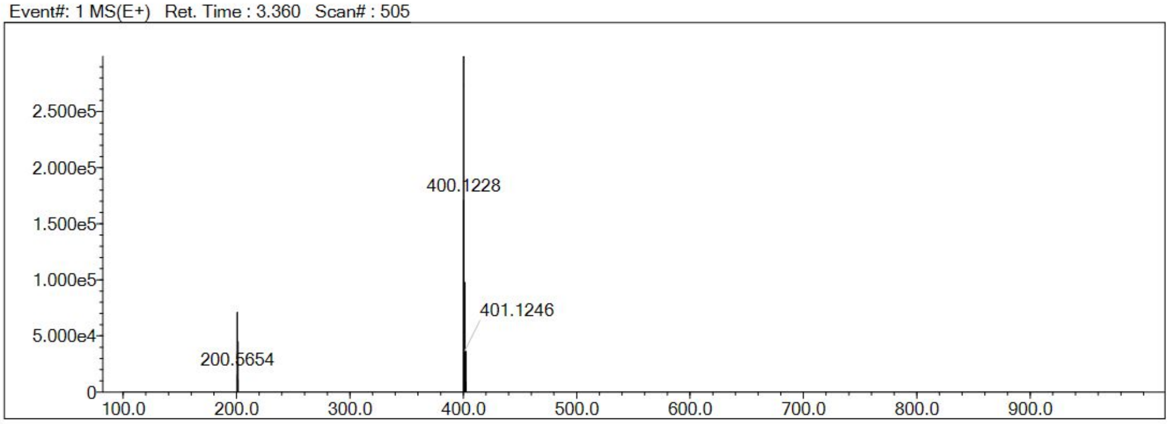

Measured region for 400.1228 m/z

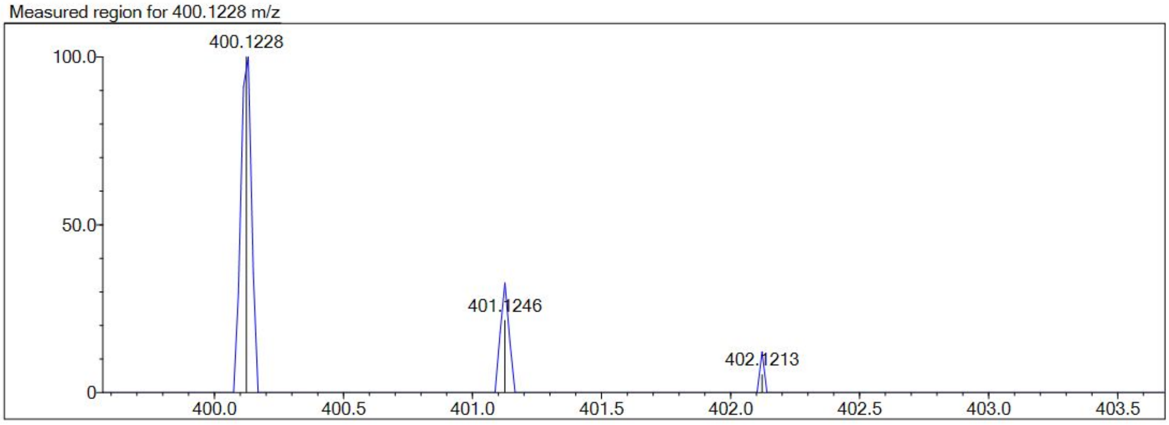

C22 H17 N5 O S [M+H]+ : Predicted region for 400.1227 m/z

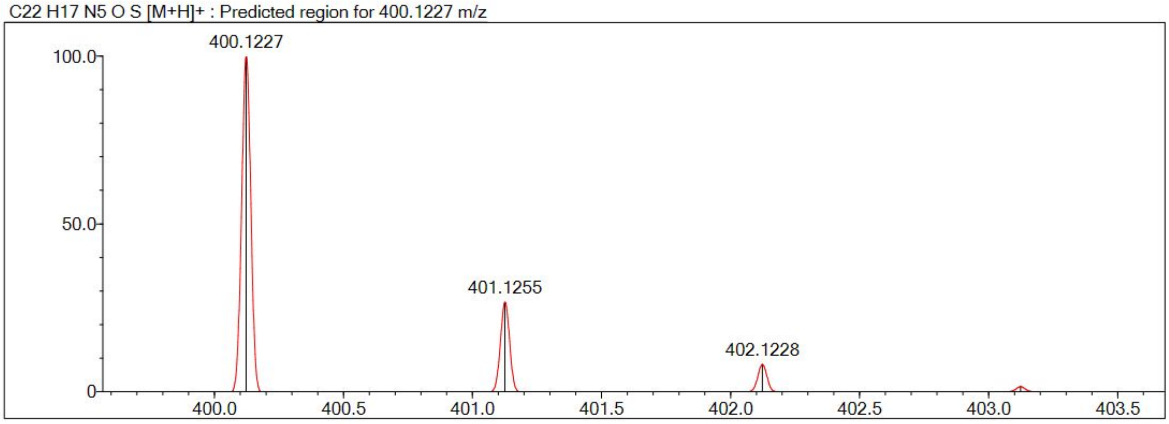

Rank	Score	Formula (M)	Ion	Meas. m/z	Pred. m/z	Df. (mDa)	Df. (ppm)	Iso	DBE
1	77.74	C22 H17 N5 O S	[M+H]+	400.1228	400.1227	0.1	0.25	77.74	17.0

Figure S21. Mass spectrum of compound **5h**

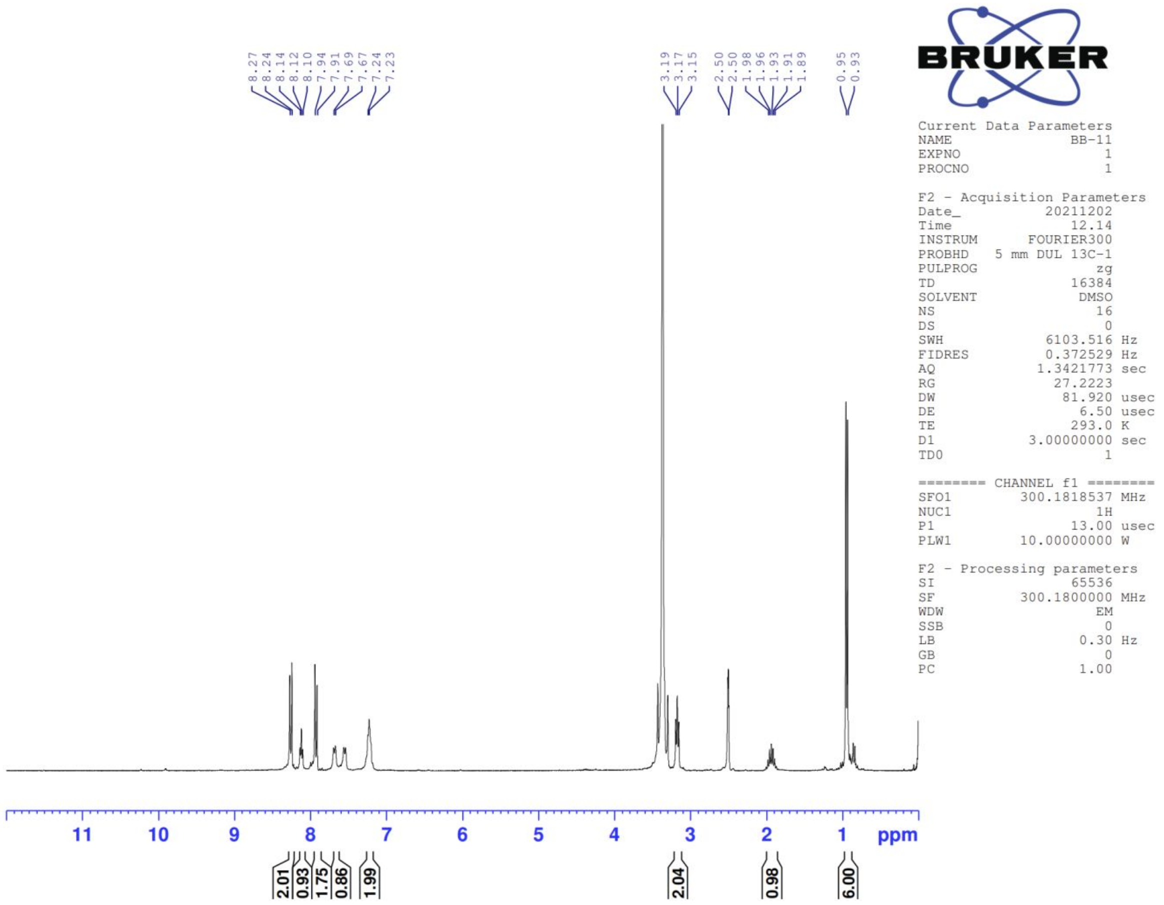

Figure S22. ^{1}H-NMR spectrum of compound **5i**

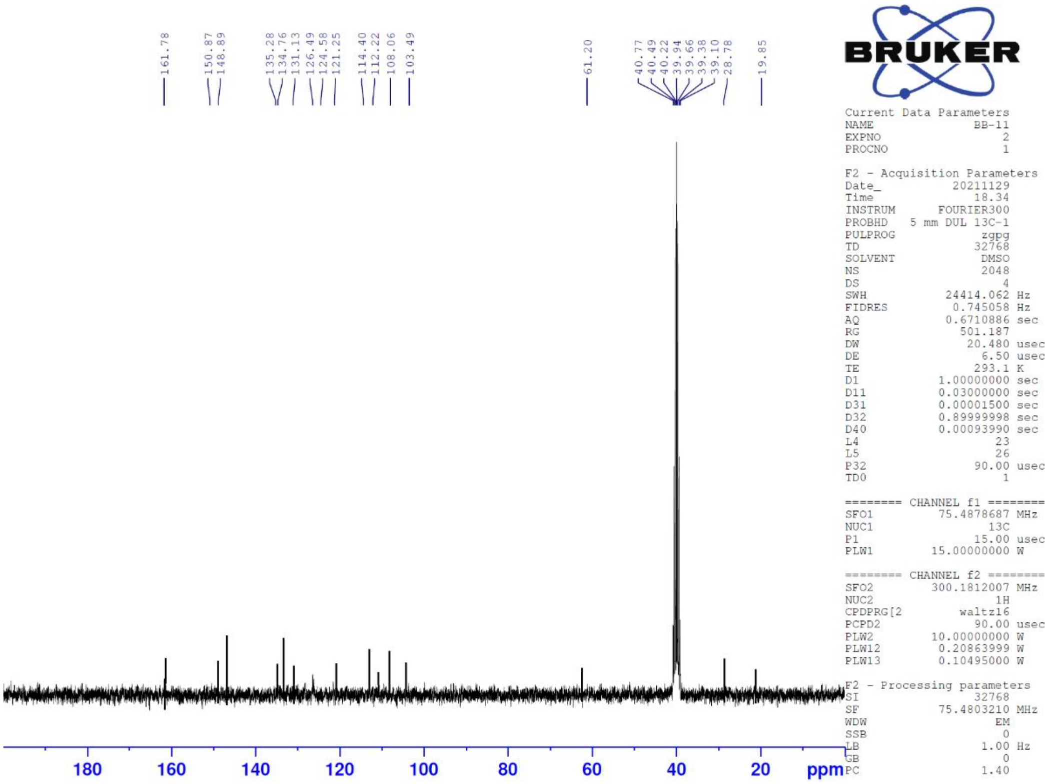

Figure S23. ^{13}C-NMR spectrum of compound **5i**

Data File: C:\LabSolutions\Data\Analiz\uac\BB-11_69.lcd

Elmt	Val.	Min	Max	Elmt	Val.	Min	Max	Elmt	Val.	Min	Max	Elmt	Val.	Min	Max	Use Adduct
H	1	10	40	O	2	0	4	S	2	1	1	Ru	2	0	0	H
C	4	9	40	F	1	0	0	Cl	1	0	0	Pd	2	0	0	
N	3	2	8	P	3	0	0	Br	1	0	0	I	3	0	0	

Error Margin (ppm):	7	DBE Range:	5.0 - 25.0	Electron Ions:	both
HC Ratio:	unlimited	Apply N Rule:	yes	Use MSn Info:	yes
Max Isotopes:	3	Isotope RI (%):	1.00	Isotope Res:	9000
MSn Iso RI (%):	10.00	MSn Logic Mode:	AND	Max Results:	150

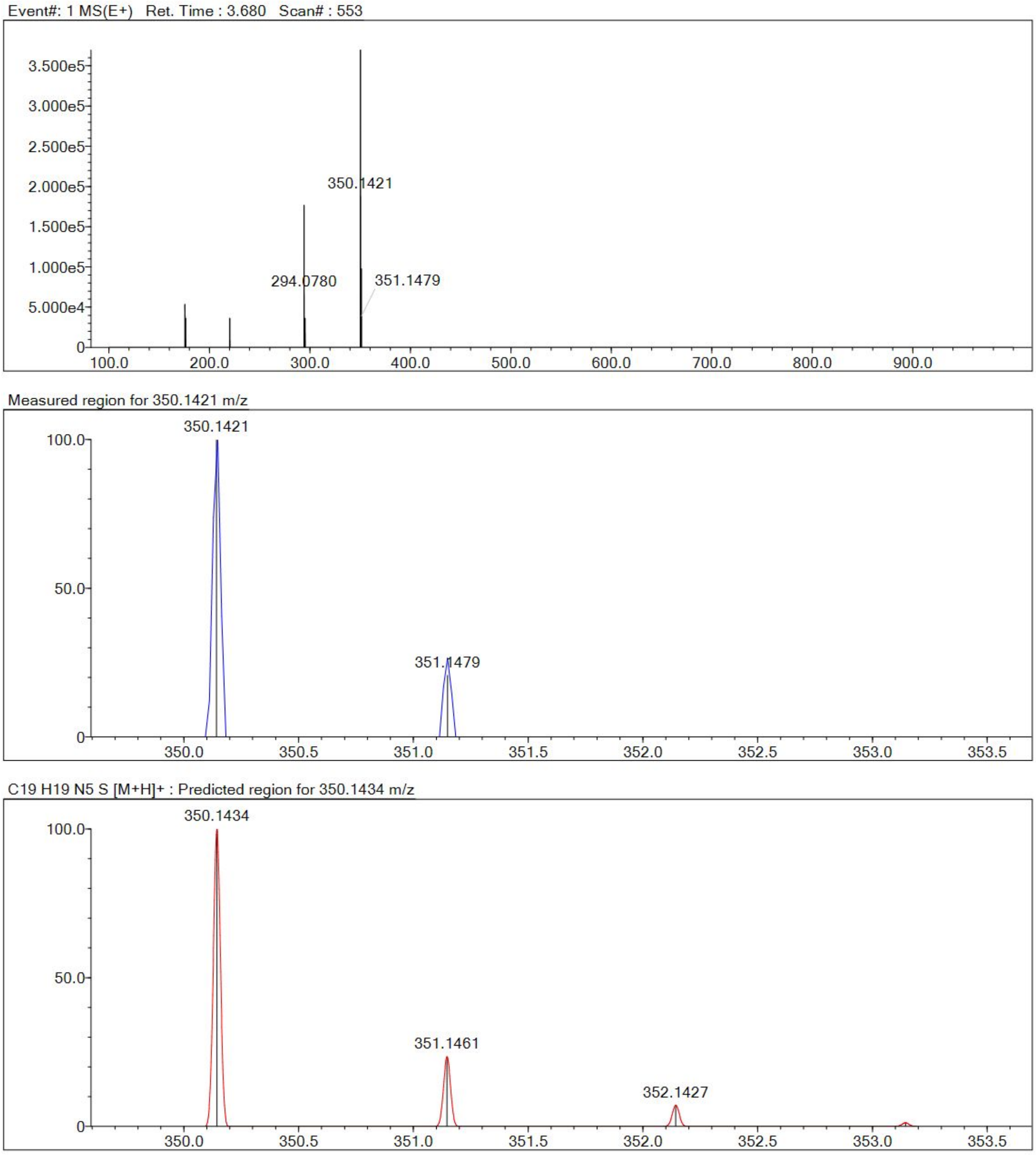

Rank	Score	Formula (M)	Ion	Meas. m/z	Pred. m/z	Df. (mDa)	Df. (ppm)	Iso	DBE
1	0.00	C19 H19 N5 S	[M+H]+	350.1421	350.1434	-1.3	-3.71	0.00	13.0

Figure S24. Mass spectrum of compound **5i**
